# Scroll-Wave Dynamics in Human Cardiac Tissue: Lessons from a Mathematical Model with Inhomogeneities and Fiber Architecture

**DOI:** 10.1371/journal.pone.0018052

**Published:** 2011-04-05

**Authors:** Rupamanjari Majumder, Alok Ranjan Nayak, Rahul Pandit

**Affiliations:** 1 Department of Physics, Centre for Condensed Matter Theory, Indian Institute of Science, Bangalore, India; 2 Jawaharlal Nehru Centre for Advanced Scientific Research, Bangalore, India; Queensland Institute of Medical Research, Australia

## Abstract

Cardiac arrhythmias, such as ventricular tachycardia (VT) and ventricular fibrillation (VF), are among the leading causes of death in the industrialized world. These are associated with the formation of spiral and scroll waves of electrical activation in cardiac tissue; single spiral and scroll waves are believed to be associated with VT whereas their turbulent analogs are associated with VF. Thus, the study of these waves is an important biophysical problem. We present a systematic study of the combined effects of muscle-fiber rotation and inhomogeneities on scroll-wave dynamics in the TNNP (ten Tusscher Noble Noble Panfilov) model for human cardiac tissue. In particular, we use the three-dimensional TNNP model with fiber rotation and consider both conduction and ionic inhomogeneities. We find that, in addition to displaying a sensitive dependence on the positions, sizes, and types of inhomogeneities, scroll-wave dynamics also depends delicately upon the degree of fiber rotation. We find that the tendency of scroll waves to anchor to cylindrical conduction inhomogeneities increases with the radius of the inhomogeneity. Furthermore, the filament of the scroll wave can exhibit drift or meandering, transmural bending, twisting, and break-up. If the scroll-wave filament exhibits weak meandering, then there is a fine balance between the anchoring of this wave at the inhomogeneity and a disruption of wave-pinning by fiber rotation. If this filament displays strong meandering, then again the anchoring is suppressed by fiber rotation; also, the scroll wave can be eliminated from most of the layers only to be regenerated by a seed wave. Ionic inhomogeneities can also lead to an anchoring of the scroll wave; scroll waves can now enter the region inside an ionic inhomogeneity and can display a coexistence of spatiotemporal chaos and quasi-periodic behavior in different parts of the simulation domain. We discuss the experimental implications of our study.

## Introduction

Cardiac arrhythmias, such as ventricular tachycardia (VT) and ventricular fibrillation (VF), are among the leading causes of death in the industrialized world. There is growing consensus [1]–[7] that such arrhythmias are associated with the formation of spiral and scroll waves of electrical activation in mammalian cardiac tissue; single spiral and scroll waves are believed to be associated with VT whereas their broken, turbulent analogs are thought to be the underlying cause of VF. Thus, the study of these waves and their eventual elimination from cardiac tissue is a problem of central importance in biomedical and biophysical science. The existence of spiral and scroll waves in mammalian cardiac tissue has been confirmed by a variety of *in vitro* and *in vivo* studies as reported, e.g., in Refs. [8]–[17]; and these have been complemented by *in silico* investigations of spiral- and scroll-wave dynamics in mathematical models of cardiac tissue [1]–[3], [18]–[24]. *In silico* studies have a significant advantage over experimental ones when we consider rotating scroll waves or scroll-wave turbulence in three-dimensional cardiac tissue because we can visualize scroll-wave dynamics in three-dimensional computational domains in much more detail than is possible experimentally. Furthermore, we can tune parameters, such as the Calcium-channel conductance, the degree of muscle-fiber rotation, or the position or the size of a conduction inhomogeneity, with ease, and thus explore their effects on scroll waves. A detailed understanding of such effects is of paramount importance in controlling scroll-wave turbulence by low-amplitude electrical stimuli; the development of such control methods is the computational analog of low-amplitude defibrillation.

Strictly speaking a spiral wave is broken at its tip, and, in 3D, a scroll wave at its filament and the association of a single spiral wave with VT and multiple spiral waves with VF, is one possible scenario for these arrhythmias; VT can also exist because of ectopic activity and VF can arise from a chaotically drifting spiral wave, but without any further breakup.

Inhomogeneities in cardiac tissue affect spiral and scroll waves in many different ways. Experimental studies [25]–[27] have shown that conduction inhomogeneities can either lead to the anchoring or pinning of such waves or they can, occasionally, eliminate these waves completely [28]. The larger the obstacle the higher is the tendency of anchoring [25]–[27]; but, in some cases, wave pinning might not occur even if the obstacle is large; and such waves can get attached to small conduction inhomogeneities, but only infrequently [27].

Numerical simulations have also been used to investigate the effects of a variety of inhomogeneities on spiral-wave dynamics in mathematical models for cardiac tissue: For example, Ref. [29] contains an investigation of spiral waves in the two-dimensional (2D) Luo-Rudy Phase I (LRI) model in an annular domain, with parameters and initial conditions such that, in the absence of the inner hole of the annulus, spiral waves break up. As the radius of the hole is reduced, from 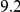 cm to 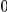, the simulation domain goes from a 1D ring to a 2D annulus, and, finally, to a homogeneous 2D domain: When the system is a 1D ring the wave goes around it; in the annular case the wave is a spiral pinned to the inner hole, if its radius is sufficiently large; as this radius decreases transitions occur first to a quasi-periodically rotating spiral wave and, eventually, to spiral-wave break up and spatiotemporal chaos [29] with the spirals not attached to the hole.

Reference [30] has studied the effects of a random distribution of non-excitable cells spiral waves in the Panfilov model; as the percentage of such non-excitable cells increases spiral-wave break up is suppressed.

Analytical and numerical studies of the interplay of spiral waves with a piecewise-linear obstacle appear in Ref. [31]. The principal results of this are the following: if the medium has a high excitability, a wave can go around the boundary of the inhomogeneity and continue thereafter as if no obstacle had been encountered; in the low-excitability case, the two free ends survive because they cannot rejoin beyond the obstacle so they develop into spiral waves that can break up subsequently; and, in addition to the excitability and wave front's local curvature, the shape of the inhomogeneity also affects the attachment of spiral waves to it.

Earlier studies from our group [2], [3] have presented numerical investigations of spiral-wave dynamics in the presence of conduction and ionic inhomogeneities in the Panfilov, LRI, reduced-Priebe-Beuckelmann, and TNNP models in two-dimensional simulation domains; preliminary studies of scroll-wave interactions with such inhomogeneities have also been considered in three-dimensional simulation domains for the simple Panfilov model without fiber rotation. The principal qualitative result of these studies is that the dynamics of spiral waves depends very sensitively on the position, shape, size, and type (i.e., conduction or ionic) of inhomogeneity; for details we refer the reader to Refs. [2], [3]; ionic inhomogeneities also lead to anchored spiral waves, but with dynamics that is considerably richer than in the case of conduction obstacles; e.g., we have found rotating spiral waves with period-4 and period-5 cycles and also states in which there is a coexistence of a periodically rotating spiral and with broken spiral waves, in the region of the ionic inhomogeneity. Our studies of the effects of inhomogeneities on scroll waves in mathematical models for cardiac tissue had not included fiber rotation in 3D simulation domains. This is the problem we address here.

Studies by several groups [20], [23], [32]–[36] have shown that fiber rotation plays an important role in the spatiotemporal evolution of scroll waves in mathematical models of cardiac tissue. We summarize the results of these studies before we embark on our study of scroll waves with *both* fiber rotation and inhomogeneities. These studies have investigated the effects of fiber rotation on scroll-wave dynamics in simple two-variable, FitzHugh-Nagumo-type models and also in ionic models such as the LRI and TNNP models.

The intramural rotation of cardiac muscle fibers makes wave propagation in ventricular tissue very anisotropic as noted, e.g., in Ref. [32] for simple FitzHugh-Nagumo-type models and for the Beeler-Reuter model. This anisotropy can twist scroll-wave filaments and thus modify their dynamics; if this twist is very large it can destabilize a single transmural filament, create vortex rings, which can, in turn, expand and create new filaments that move chaotically. These fibers also lead to a propagation anisotropy [20]: the conduction velocity along the fiber axis is approximately three times greater than it is across it. This anisotropy can lead to a twisting of scroll-wave filaments and break up of scroll waves even if the excitability is not low [37]–[39]. In Ref. [36] the effects of fiber rotation on scroll-wave dynamics have been studied numerically for the LRI model but without any inhomogeneities; with the original LRI-model parameters, these studies find that a minimum thickness is required for scroll-wave break-up, which proceeds via filament bending. The effects of the transmural rotation of fibers on scroll waves have also been studied for the LRI model in cubical domains [40] of thickness 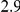 mm particularly with a view to elucidating the effects of different fiber-rotation rates on the dynamics of these waves; as this rate increases the susceptibility of the medium to re-entry decreases but complex spatiotemporal patterns can occur. The work of Ref. [35] elucidates the differences between the dynamics of scroll waves in cuboid and anatomically realistic simulation domains; this study includes fiber rotation, uses the TNNP model, but does not include inhomogeneities of the type we study here.

Our earlier work [2], [3] has elucidated the effects of different types of inhomogeneities – conduction and ionic – on spiral- and scroll-wave dynamics in mathematical models of cardiac tissue *but without fiber rotation*. The studies we have summarized above have studied the effects of fiber rotation on scroll-wave dynamics on a variety of mathematical models for cardiac tissue *but without conduction or ionic inhomogeneities*. To the best of our knowledge, the study we present here is the first one to investigate the *combined* effects of fiber rotation and inhomogeneities on scroll-wave dynamics in the three-dimensional, detailed ionic TNNP model (principally with epicardial parameters).

The effect of transmural heterogeneities has been studied in detail in Refs. [41]–[43], in two-dimensional simulations, and in Ref. [35] in isotropic, orthotropic and anisotropic three dimensional geometries. However, such studies do not include the role of inhomogeneities, which have a profound effect on the dynamical behavior of spiral and scroll waves. In this paper we principally investigate the effects of such inhomogeneities in the epicardium, and, in three representative cases, in the whole thickness of the ventricular wall.

Our principal goal is to study the effects of cardiac fiber rotation (FR) on the dynamics of scroll waves in three-dimensional (3D) simulation domains, both without and with inhomogeneities, in the mathematical model of cardiac tissue due to ten Tusscher, Noble, Noble, and Panfilov (TNNP) [44]. By FR we mean the following: The fibers in each plane are rotated, with respect to those in adjacent planes, by an angle 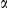; thus, the orientation of the fibers in a plane is specified by an angle 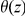 that varies continuously with the transmural coordinate 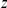. In a normal human heart the average total FR, from the endocardium to the epicardium, is 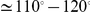. The distribution of this angle over the three-layered heart wall varies across mammalian species and, within a specie, from individual to individual. Our study here extends considerably our earlier work [3] on spiral-wave dynamics in the two-dimensional (2D) TNNP model; the principal qualitative result of this study was that such dynamics depends delicately on the positions, shapes, and sizes of conduction and ionic inhomogeneities.

In the 3D TNNP model that we study here with fiber rotation and with conduction or ionic inhomogeneities, we find the following qualitatively interesting results:

1. In addition to displaying a sensitive dependence on the positions, sizes, and types of inhomogeneities, scroll-wave dynamics also depends upon the degree of fiber rotation.

2. Scroll waves do not anchor easily to cylindrical conduction inhomogeneities with small radii; this anchoring tendency increases with the radius; this result is in agreement with the experimental findings of Refs. [27], [45]–[49]. We conjecture that our result is a three-dimensional manifestation of the findings of Ref. [50] in which the authors suggest that, if the radius of the closed trajectory of the tip of a spiral wave is less than the radius of an obstacle, then this wave is pinned to the obstacle; if not, then the wave can be removed by suitable pacing of the medium.

3. If the scroll-wave filament exhibits weak meandering, then there is a fine balance between the anchoring of this wave at the inhomogeneity and a disruption of wave-pinning by fiber rotation; by contrast, if this filament displays strong meandering, then anchoring is still suppressed by fiber rotation, but, in addition, the scroll wave can be eliminated from most of the layers only to be regenerated by a seed wave.

4. We find, furthermore, that ionic inhomogeneities, which we model by changing the values of certain conductances in a cylindrical region in our simulation domain, can also lead to an anchoring of the scroll wave; scroll waves can now enter the region inside an ionic inhomogeneity and the system can display a coexistence of spatiotemporal chaos and quasi-periodic behavior in different parts of the simulation domain.

The remaining part of this paper is organized as follows. The section entitled **Results** contains a detailed description of our results. It has two subsections: the first of these deals with our studies of the 3D TNNP model in the absence of inhomogeneities; the second subsection contains our studies of the 3D TNNP model with inhomogeneities. The section entitled **Discussions** contains a discussion of our results and the section labeled **Methods** is devoted to a description of the TNNP model and the numerical details of our simulations.

## Results

**Figure 1:**
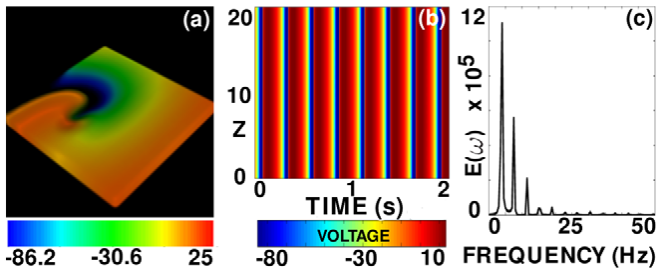
Scroll-wave dynamics in the 3D TNNP model in the absence of fiber rotation and inhomogeneities. (a) A volume rendering of the transmembrane potential 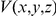, at the representative time 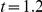
s (the spatiotemporal evolution of 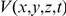 for this case is shown in Video S1. (b) A space-time 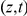 pseudocolor plot of 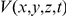 at the representative point 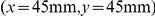. (c) The power spectrum 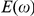 of the time series 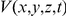 from the representative point 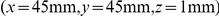; from this we see that the scroll wave rotates quasiperiodically in the medium because the principal peaks in 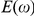 can be indexed as 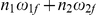, where 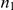 and 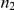 are integers and 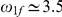
Hz and 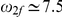
Hz are two incommensurate frequencies.

We consider first scroll-wave dynamics in a rectangular-parallelepiped simulation domain without FR or inhomogeneities in the 3D TNNP model. We begin with a simple scroll wave generated by stacking a set of spiral waves that we obtain from simulations on a two-dimensional (2D) TNNP model [3]. This simple initial scroll wave continues to rotate without significant modification of its original shape as can be seen from the volume rendering of 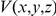, at the representative time 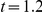
s, in figure 1(a); the spatiotemporal evolution of 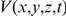 for this case is shown in Video S1 in the supplementary material. Figure 1(b) shows a space-time 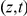 pseudocolor plot of 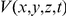 at the representative point 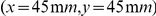. Figure 1(c) shows the power spectrum 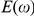 of the time series 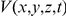 from the representative point 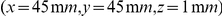; from this we see that the scroll wave rotates quasi-periodically in the medium because the principal peaks in the power spectrum 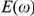 can be indexed as 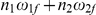, where 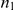 and 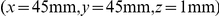 are integers and 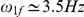 and 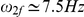 are two incommensurate frequencies.

We now investigate how the dynamical behavior of this scroll-wave changes as we introduce FR and inhomogeneities in the medium. Subsection **No inhomogeneities** deals with the investigation of scroll-wave dynamics in the absence of localized inhomogeneities. Subsection **Inhomogeneities** deals with the combined effects of FR and inhomogeneities on scroll-wave dynamics; this subsection has two parts.

### No inhomogeneities

**Figure 2:**
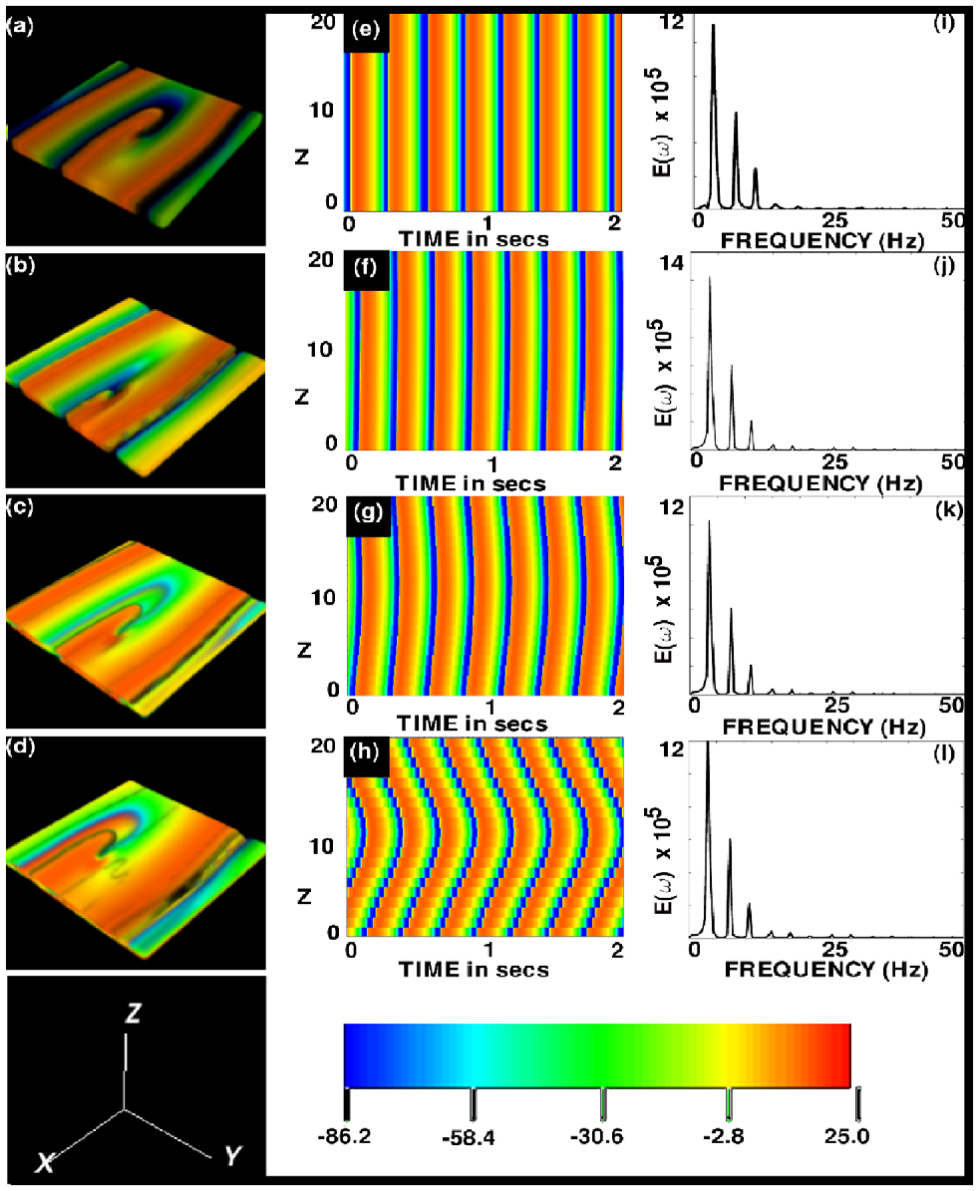
Scroll-wave dynamics in the 3D TNNP model with fiber rotation but no inhomogeneities. The volume renderings (a), (b), (c), and (d) show, at the representative time 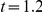
s, 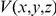 for the FR angles 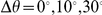 and 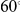, respectively (the spatiotemporal evolution of 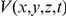 for the representative case of 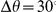 is shown in Video S2 in the supplementary material; the videos for the cases with 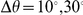 and 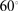 are available at http://www.physics.iisc.ernet.in/~rahul/new_movies.html). The space-time 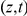 pseudocolor plots (e), (f), (g), and (h) show 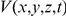, at the representative point 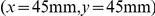 for the same FR angles; and (i), (j), (k), and (l) show power spectra 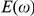 of the time series 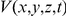 from the representative point 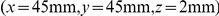.

Figures 2(a), (b), (c), and (d) show, at the representative time 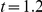
s, volume renderings of 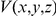 for the FR angles 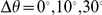 and 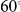, respectively; the spatiotemporal evolution of 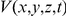 for the representative case of 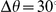 is shown in Video S2 in the supplementary material; the videos for the cases 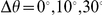 and 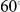, are available at http://www.physics.iisc.ernet.in/~rahul/new_movies.html. Figures 2(e), (f), (g), and (h) show space-time 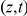 pseudocolor plots of 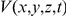, at the representative point 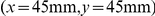 for the same values of 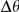; and figures 2(i), (j), (k), and (l) show the power spectra 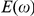 of the time series 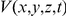 from the representative point 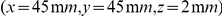. By comparing figure 1(a) with figures 2(a)-(d) (or Video S1 with the videos for the cases with 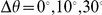 and 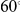) we see that the most dramatic effect of FR is a distortion of the scroll-wave relative to its form in the completely homogeneous case; this distortion arises from the difference between 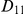 and 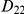 in the diffusion tensor (see the section labeled **Methods**); however, for the case with no fiber rotation, the TNNP model, with the tensor 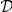, can be mapped onto the homogeneous case by suitable rescaling of the linear dimensions along and perpendicular to the fiber orientation.

**Figure 3:**
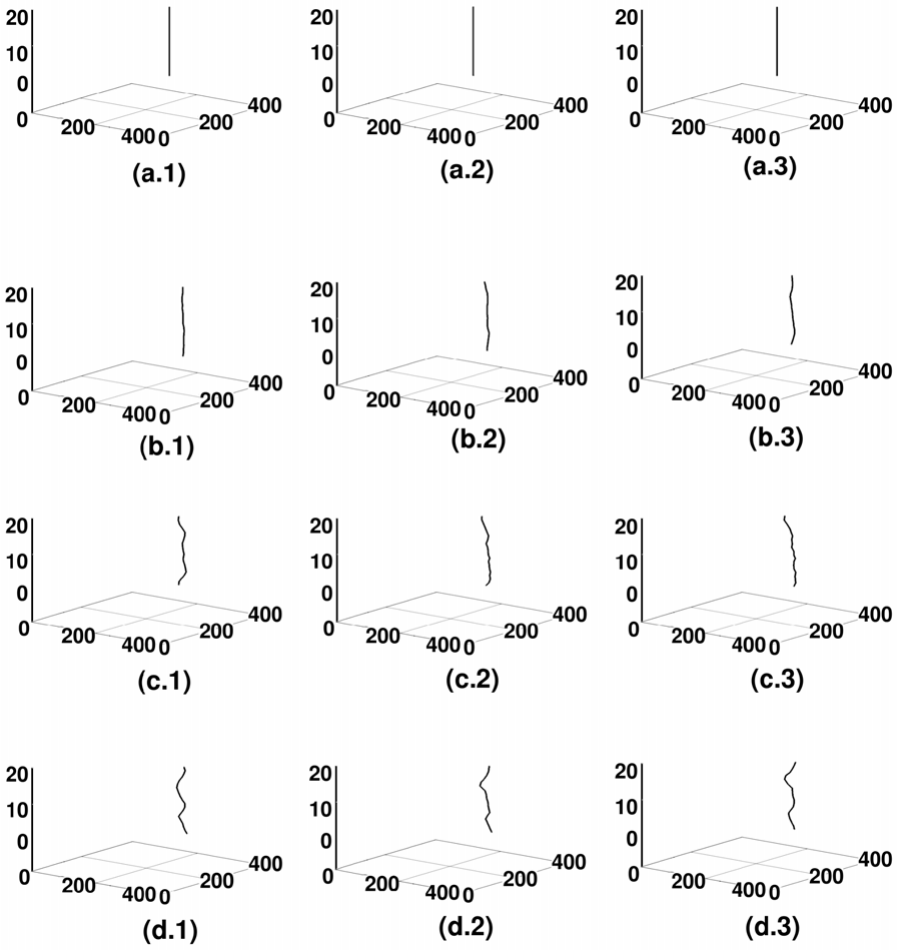
Spacetime evolution of the filament of the scroll wave in the absence of inhomogeneities with 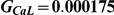 nS/pF. Spacetime evolution of the filament of the scroll wave is shown in the absence of inhomogeneities. For 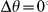, figures (a.1), (a.2), and (a.3) show, respectively, the filament of the scroll wave at 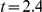
s, 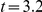
s, and 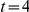
s; the analogs of these figures for 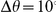 are figures (b.1), (b.2), and (b.3), for 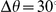 are figures (c.1), (c.2), and (c.3) and for 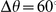 are figures (d.1), (d.2), and (d.3).

A comparison of the space-time plots in figures 2(e) and (f) and of the power spectra in figures 2(i) and (j) shows that the spatiotemporal behaviors of the scroll waves for 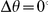 and 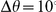 are very similar. Deviations from the behavior for 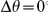 become noticeable for 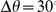 and very significant for 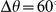; the rotation frequencies remain approximately the same [see figures 2 (i)-(l)]; but the bowing in the space-time plots of figures 2(g) and (h) indicate that, as 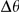 increases, the spiral sections of the scroll wave are such that the one in the central plane 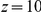 (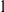 mm) leads those in the planes above and below it. This bowing is a manifestation of the transmural bending of the filamentary core of the scroll wave; and this bending can be seen clearly in Video S2, by looking at the curvature of the patterns on the boundaries that are normal to the 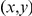 plane, and in the 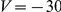 mV isosurface video, Video S3 (see supplementary material) in which we have zoomed in to the region near the core of the scroll wave. Such a bending of the scroll-wave filament has been seen earlier in a numerical study of the Luo-Rudy I (LRI) model [36] that includes FR. In the LRI model the bending of the filament increases with 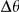, as in the TNNP model we study; but, for sufficiently large 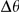, the filament of the LRI scroll wave breaks. We have not found such filament breaking in the TNNP model, with the standard parameters [44] and for the range of values of 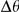 that we have used, even when we have increased the thickness of the simulation domain from 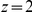 mm to 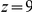 mm. For 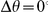, figures 3(a.1), 3(a.2), and 3(a.3) show, respectively, the filament of the scroll wave at 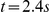, 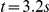, and 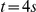; the analogs of these figures for 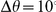 are figures 3(b.1), 3(b.2), and 3(b.3), for 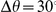 are figures 3(c.1), 3(c.2), and 3(c.3), and for 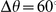 are figures 3(d.1), 3(d.2), and 3(d.3).

**Figure 4:**
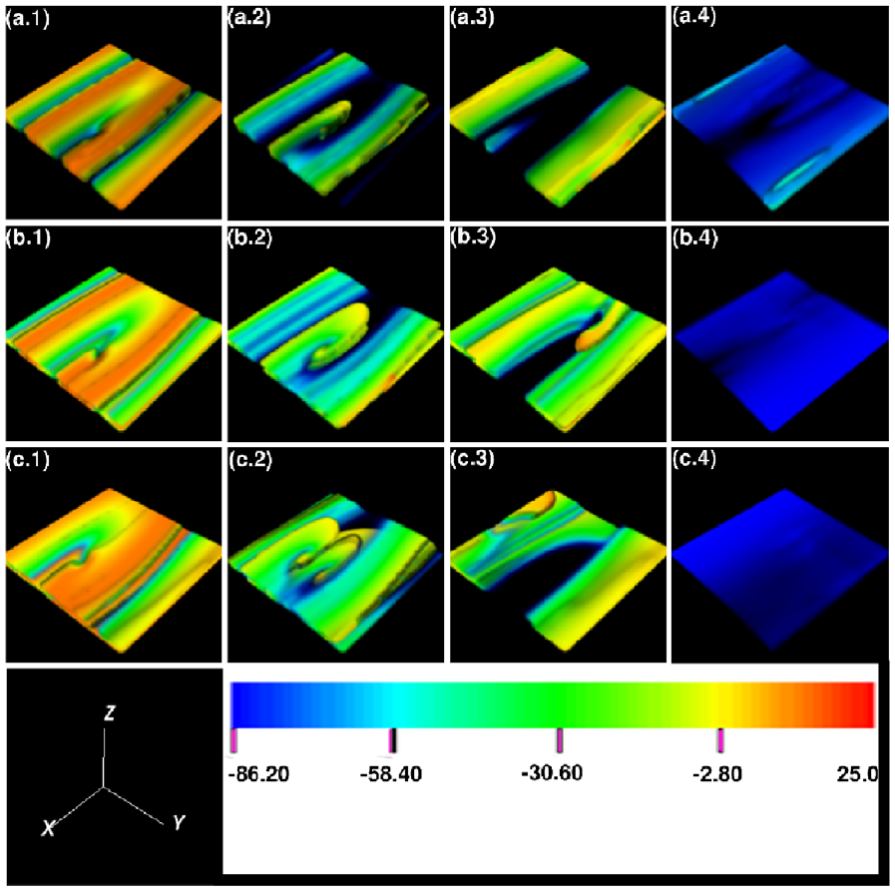
Scroll-wave dynamics in the 3D TNNP model with fiber rotation but no inhomogeneities; here the *L-type* 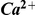 conductance 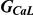 is reduced to 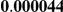 nS/pF (its normal value 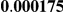 nS/pF yields the plots in figure 2). The volume renderings (a.1), (a.2), (a.3), and (a.4) show, for 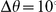, the transmembrane potential 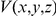 for times 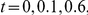 and 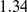
s, respectively; their analogs for 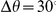 and 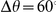 are given in (b.1)-(b.4) and (c.1)-(c.4). (The spatiotemporal evolution of 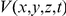 is shown, for the representative case with 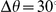, in the video files, Video S4 and Video S5 in the supplementary material; a comparison of Video S4 with Video S2, in which 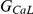 has its standard value, shows clearly the difference between scroll waves that break up and those that do not.).

**Figure 5:**
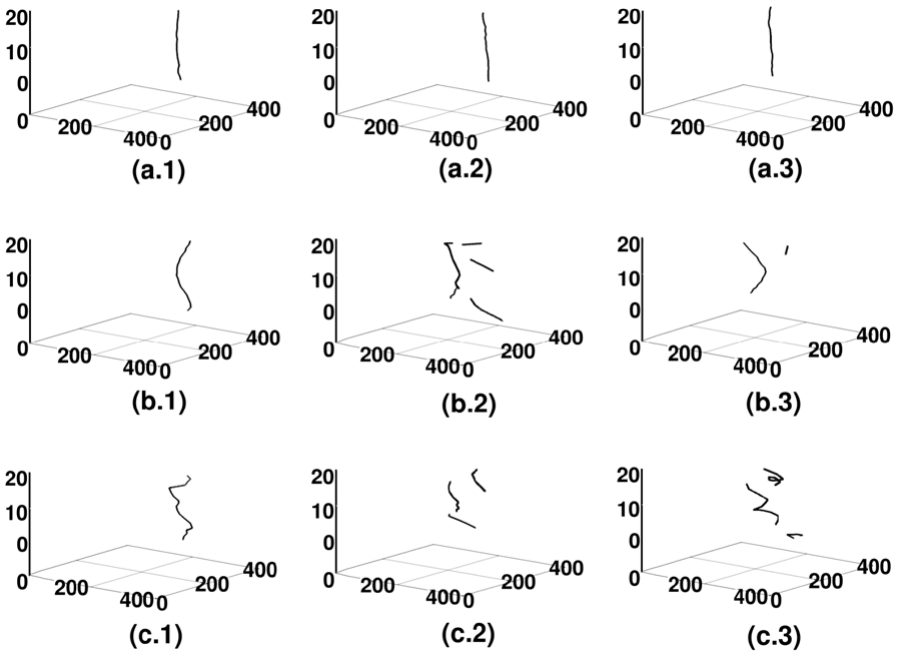
Spacetime evolution of the filament of the scroll wave in the absence of inhomogeneities with 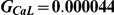 nS/pF. For 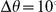, figures (a.1), (a.2), and (a.3) show, respectively, the filament of the scroll wave at 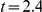
s, 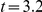
s, and 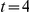
s; the analogs of these figures for 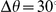 are figures (b.1), (b.2), and (b.3), and for 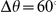 are figures (c.1), (c.2), and (c.3).

**Figure 6:**
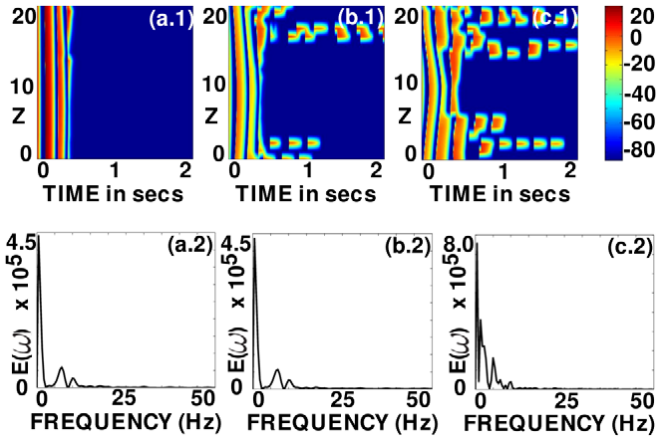
Scroll-wave dynamics in the 3D TNNP model with fiber rotation but no inhomogeneities; here the *L-type* 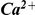 conductance 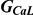 is reduced to 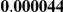 nS/pF (its normal value 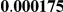 nS/pF yields the plots in figure 2). (a.1), (b.1), and (c.1) show space-time 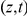 pseudocolor plots of 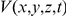, at the representative point 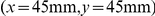, for the FR angles 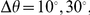 and 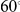, respectively; and (a.2), (b.2), and (c.2) show the power spectra 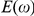 obtained from the time series 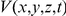 from the representative point 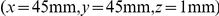.

We find, however, that the 3D TNNP model does show scroll-wave break-up if we change some parameters. In particular, if (a) we reduce the *L-type* 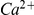 conductance 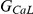 to one-fourth of its maximum value or (b) we increase the plateau 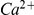 conductance 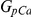 by a factor of 5, we obtain such break-up. In Figure 4 we illustrate the spatiotemporal evolution of a scroll wave when the *L-type* 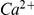 conductance has been reduced; to start with, we have a single rotating scroll wave with a straight filament; as time passes, for low 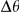 (say 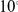), the filament of the scroll wave does not break up but it drifts away from the center of the simulation domain; finally it hits the boundaries where it is absorbed. For intermediate to high values of 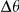 (say 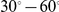), the filament of the scroll wave meanders, breaks and leads to a state characterized by scroll-wave turbulence; finally, however, the broken scroll waves are absorbed at the boundaries and the simulation domain is free of scroll waves. Figures 4(a.1), (a.2), (a.3), and (a.4) show, for 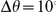, volume renderings of 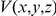 for times 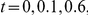 and 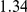
s, respectively; their analogs for 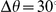 and 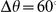 are given in figures 4(b.1)-(b.4) and figures 4(c.1)-(c.4). The spatiotemporal evolution of 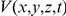 is shown, for the representative case with 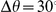, in the video files, Video S4 and Video S5 of the supplementary material; a comparison of Video S4 with Video S3, in which 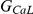 has its standard value, shows clearly the difference between scroll waves that break up and those that do not. The behaviors of the scroll-wave-filaments is illustrated in figure 5. For 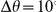, figures 5(a.1), 5(a.2), and 5(a.3) show, respectively, the filament of the scroll wave at 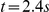, 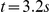, and 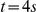; the analogs of these figures for 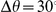 are figures 5(b.1), 5(b.2), and 5(b.3), and for 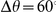 are figures 5(c.1), 5(c.2), and 5(c.3). Figures 6(a.1), (b.1), and (c.1) show space-time 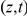 pseudocolor plots of 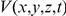 corresponding to figure 4, at the representative point 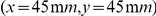, for the FR angles 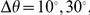 and 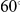, respectively; and figures 6(a.2), (b.2), and (c.2) show the power spectra 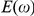 obtained from the time series 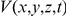 from the representative point 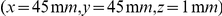.

**Figure 7:**
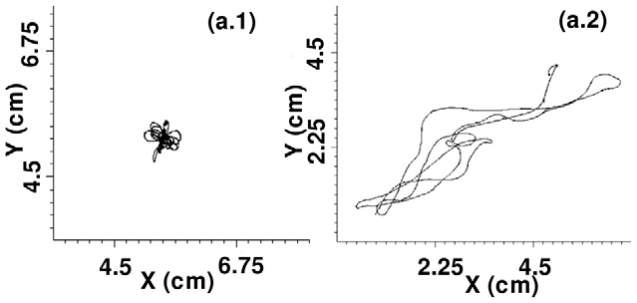
Representative tip-trajectories of the spiral wave that is obtained by taking a section at 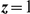 mm through our simulation domain, for the case with 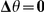. In (a.1), which uses 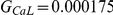 nS/pF, the meandering of the tip is limited to a region with linear dimension 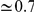 cm, as is expected in a weak-meander régime; in (a.2), which uses 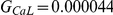 nS/pF, the tip meanders considerably as is to be expected in the régime of strong meander.

Figure 7 illustrates the spatiotemporal evolution of the tip of the spiral wave, which is obtained by taking a section at 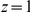 mm through our simulation domain, for the case with 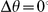. In the first sub-figure, namely 7(a.1), which uses the original value of 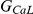, the meandering of this tip is limited to a region with linear dimension 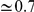 cm, as is expected in a weak-meander régime; in the second of these sub-figures, namely 7(a.2), which uses the modified value of 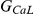, the tip meanders considerably as is to be expected in the régime of strong meander. Hence we refer to the respective parameter régimes as the *weak meander régime* and the *strong meander régime* in the remaining part of our paper. In most of our simulations, we use a simulation domain of size 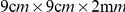. We have carried out a few representative studies on a simulation domain of size 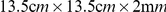 that contains 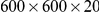 grid points. We find that, in cases where broken scroll waves are eventually absorbed by boundaries, these waves survive longer in large simulation domains than they do in small ones.

**Figure 8:**
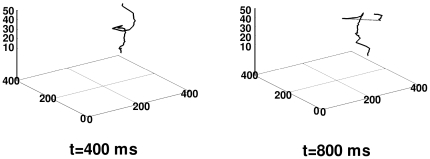
Scroll-wave filament in the 3D TNNP model with transmural heterogeneity and fiber rotation. Representative plots, at time 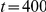 and 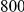 ms of the filament of a scroll wave in the 3D TNNP with transmural heterogeneity and fiber rotation; at 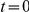 we start with a scroll wave whose filament is straight; the widths of the epicardial, mid-myocardial, and endocardial layers are, respectively, 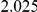 mm, 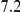 mm, and 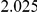 mm.

If we include transmural heterogeneity in the medium, i.e., our simulation region has epicardial, mid-myocardial, and endocardial domains as we have described in the section entitled **Methods**, the initially straight filament of the scroll wave is gradually distorted: it bends and twists and finally breaks off at the boundary between the epicardium and the mid-myocardium. We show this in the representative filament-tracking plots in figure 8.

### Inhomogeneities

We turn now to a systematic study of scroll-wave dynamics in the 3D TNNP model with FR *and* inhomogeneities; we consider both conduction and ionic inhomogeneities. As we show below, scroll waves can now exhibit rich dynamical behaviors.

### Conduction inhomogeneities

We consider cylindrical conduction inhomogeneities, which we obtain by setting all components of the diffusion tensor equal to a small number (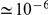) in a cylindrical region whose axis is parallel to the 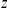 direction and that spans the thickness of our simulation domain. If the radius 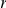 of this cylindrical obstacle is small (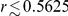 cm) the scroll wave separates into two parts when it impinges on the obstacle; these parts sweep around it, rejoin each other beyond it, and emerge finally as a plane wave; thus, the scroll wave does not get anchored to such thin, cylindrical obstacles. Fiber rotation works against such anchoring because it makes different scroll-wave layers, perpendicular to the 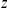 axis, rotate at different phases and move with different conduction velocities (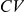
s): if in one layer the spiral-wave projection of the scroll wave gets anchored successfully to the inhomogeneity, then the spiral-wave projections in other layers are pulled away from it. In two-dimensional planar sections of the scroll wave we see spiral waves; in some layers of the simulation domain these rotate together, whereas in others they rotate out of phase with the wave in some given layer. Furthermore, if we view the scroll wave in three dimensions, we find that its filament distorts in such a way that it remains attached to the obstacle at more than one point along its length in the transmural direction; the spiral sections of such a scroll wave appear pinned to the obstacle in some layers and detached from it in other layers.

Qualitatively different dynamical behaviors are obtained if we increase the radius 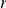 of the cylindrical conduction inhomogeneity. To illustrate such behaviors we now consider inhomogeneities with 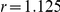 cm. We find that the dynamics of the scroll wave depends sensitively on the position and size of the obstacle, as in our earlier studies without FR [2], [3]; furthermore, this dynamics also depends sensitively on the FR angle 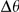: If we fix the position of the obstacle, then the dynamical response of the scroll wave depends on 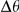; if we fix 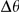, then the dynamical response of the scroll wave depends on the position of the obstacle.

Earlier studies of spiral-wave dynamics in two-dimensional mathematical models for cardiac tissue [36] have distinguished between *weak-meander* and *strong-meander* régimes. In the former, the filament of the scroll wave (or the tip of the spiral wave in a 2D simulation [3]) does not wander significantly in the simulation domain; in the latter, this filament or tip wandering is significant.

In the sections that follow, we present our results for weak- and strong-meander régimes separately. The parameters used in the original TNNP model [3], [44] lead to weak meandering; when we reduce 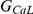 to one-fourth of its maximal value we obtain the strong-meander régime in the TNNP model.

### Weak-meander régime

**Figure 9:**
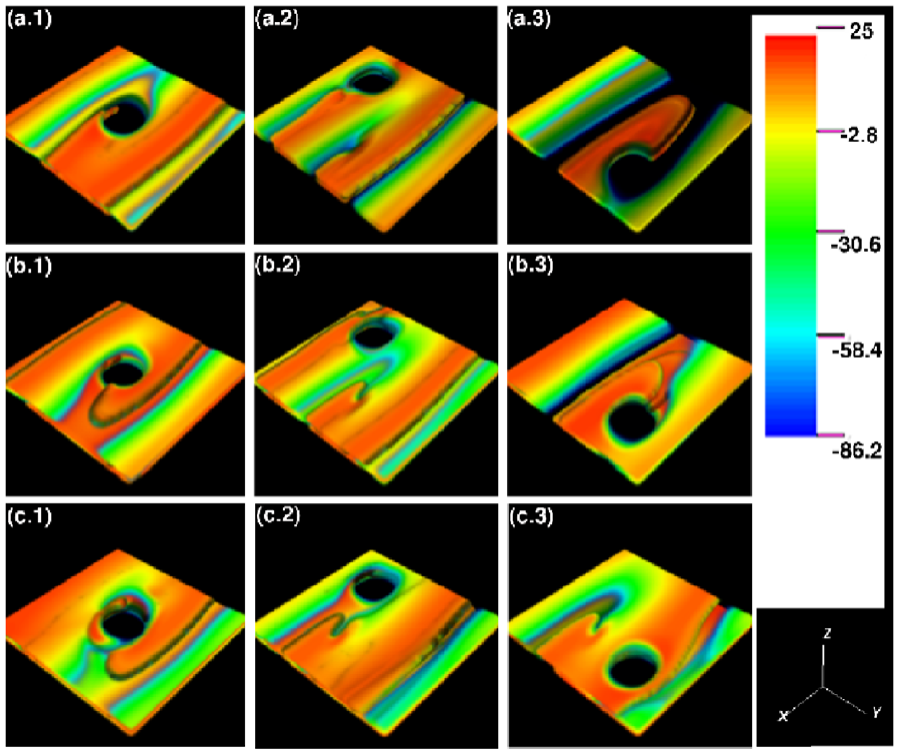
Scroll-wave dynamics in the 3D TNNP model with fiber rotation and a cylindrical conduction inhomogeneity with radius 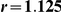 cm. Obstacle at the center of the domain: the scroll wave anchors to it for 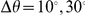, and 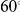, as shown in the representative volume renderings of 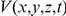 in (a.1), (b.1), and (c.1), respectively (the spatiotemporal evolution of 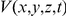 for the representative case of 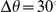 is shown in Video S6 in the supplementary material; the videos for the cases with 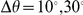 and 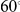 are available at http://www.physics.iisc.ernet.in/~rahul/new_movies.html). Obstacle at a corner, far from the core of the scroll: the scroll wave does not get anchored to the inhomogeneity, as shown in the representative volume renderings of 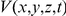 in (a.2), (b.2), and (c.2) for 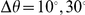, and 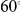, respectively (the spatiotemporal evolution of 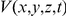 for the representative case of 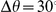 is shown in Video S7 in the supplementary material); the videos for the cases with 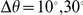 and 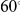 are available at http://www.physics.iisc.ernet.in/~rahul/new_movies.html). Obstacle at a corner that is close to the core of the scroll wave: distinct wave-pinning occurs at 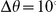 and 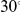, as illustrated in the representative volume renderings of 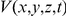 in (a.3) and (b.3). However, if 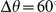, some sections of the wave readily anchor to the inhomogeneity whereas others sweep past it, reassemble beyond it, and emerge as plane waves as shown in the volume rendering of 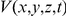 in (c.3) (the spatiotemporal evolution of 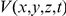 for the representative case of 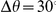 is shown in Video S8 in the supplementary material); the videos for the cases with 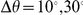 and 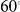 are available at http://www.physics.iisc.ernet.in/~rahul/new_movies.html).

When the obstacle is located at the center of the domain, the scroll wave anchors to it for 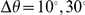, and 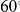, as shown in the representative volume renderings of 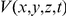 in figures 9(a.1), (b.1), and (c.1), respectively; the spatiotemporal evolution of 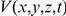 for the representative case of 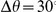 is shown in Video S6 of the supplementary material; the corresponding videos for the cases with 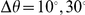, and 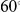 are available at http://www.physics.iisc.ernet.in/~rahul/new_movies.html.

If the obstacle lies at a corner, far from the core of the scroll, we find that the scroll wave does not get anchored to the inhomogeneity, as shown in the representative volume renderings of 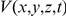 in figures 9(a.2), (b.2), and (c.2) for 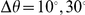, and 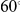, respectively; the spatiotemporal evolution of 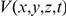 for the representative case of 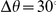 is shown in Video S7 of the supplementary material; the corresponding videos for the cases with 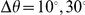, and 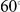 are available athttp://www.physics.iisc.ernet.in/~rahul/new_movies.html. If we shift the obstacle position to a corner that is close to the core of the scroll wave, distinct wave-pinning occurs at 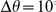 and 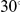, as illustrated in the representative volume renderings of 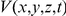 in figures 9(a.3) and 9(b.3); the spatiotemporal evolution of 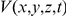 for the representative case of 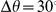 is shown in Video S8 of the supplementary material. However, if 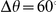, the delicate balance between anchoring at the inhomogeneity and the FR-induced disruption of wave-pinning is upset: some sections of the wave readily anchor to the inhomogeneity whereas others sweep past the obstacle, reassemble beyond it, and emerge as plane waves; this is shown in the representative volume rendering of 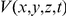 in figure 9(c.3). The spatiotemporal evolution of 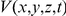 for the cases with 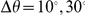, and 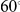 are shown in videos, available at http://www.physics.iisc.ernet.in/~rahul/new_movies.html.

**Figure 10:**
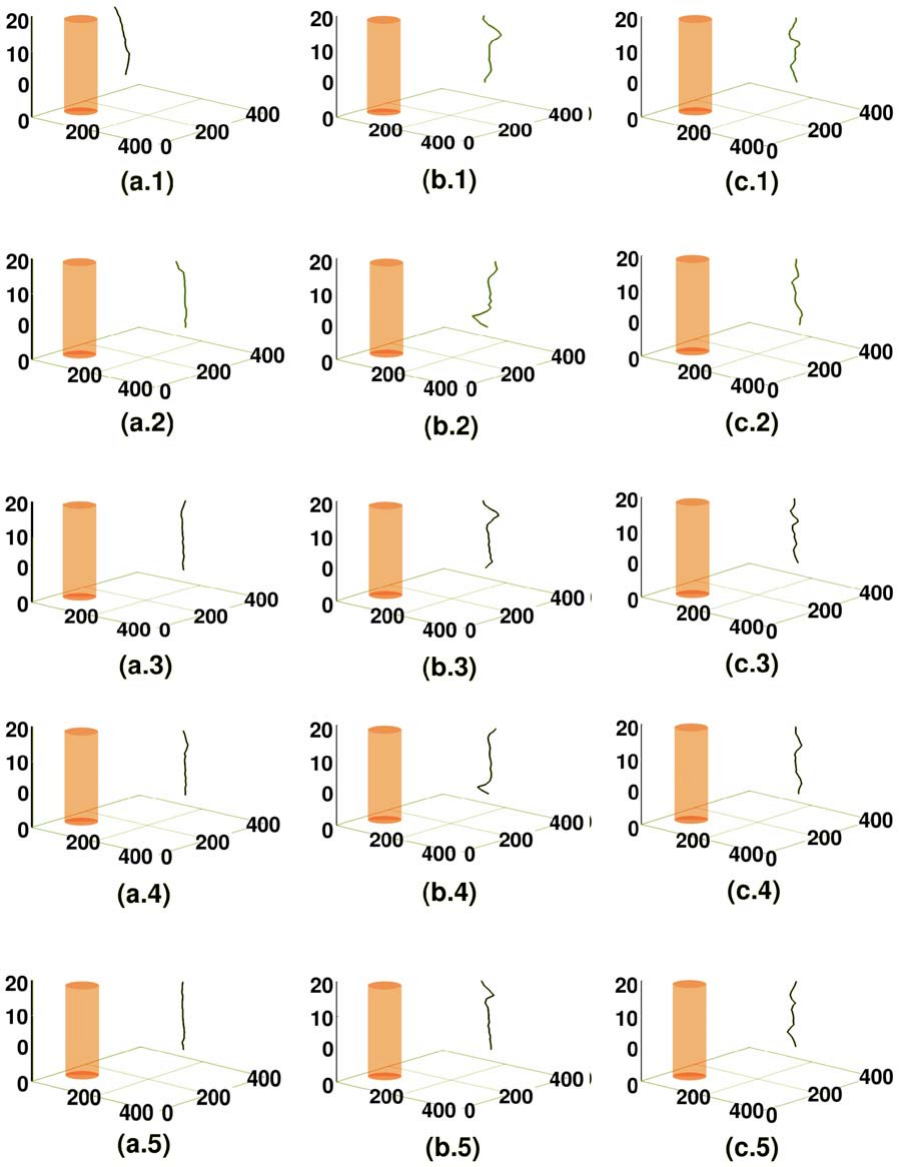
Spacetime evolution of the filament of the scroll wave in the presence of a cylindrical, conduction-type inhomogeneity located at a corner of the simulation domain that was initially far from the core of the scroll wave. These simulations use 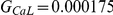 nS/pF. For 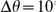, figures (a.1), (a.2), (a.3), (a.4) and (a.5) show, respectively, the filament of the scroll wave at 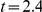
s, 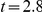
s, 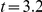
s, 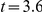
s, and 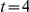
s; the analogs of these figures for 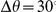 are figures (b.1), (b.2), (b.3), (b.4), and (b.5), and for 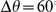 are figures (c.1), (c.2), (c.3), (c.4), and (c.5). In all of the above cases, the presence of the obstacle does not affect the dynamics of the scroll wave. The filament, which is located away from the obstacle in each case, displays bending and twisting, but no breakage.

**Figure 11:**
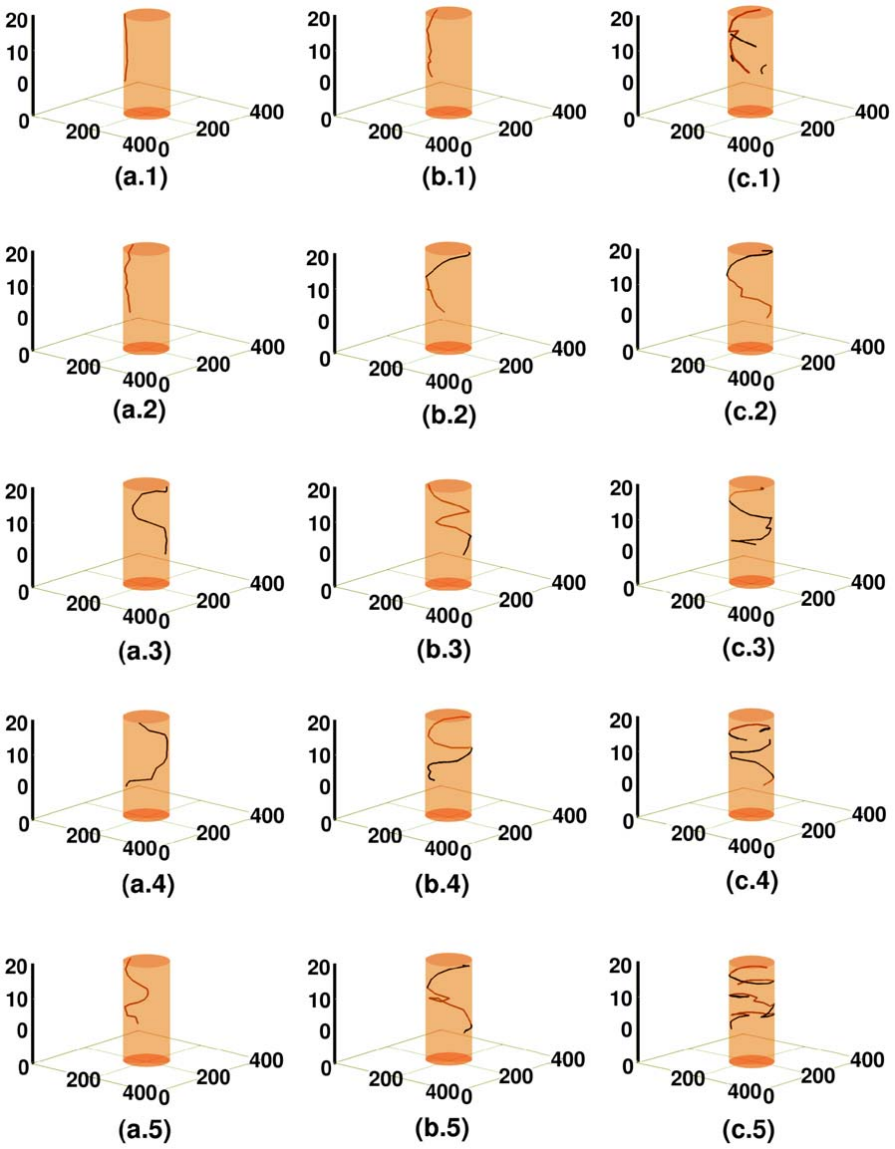
Spacetime evolution of the filament of the scroll wave in the presence of a cylindrical, conduction-type inhomogeneity located at the center of the simulation domain. These simulations use 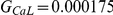 nS/pF. For 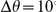, figures (a.1), (a.2), (a.3), (a.4) and (a.5) show, respectively, the filament of the scroll wave at 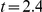
s, 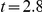
s, 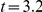
s, 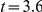
s and 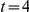
s. The analogs of these figures for 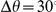 are figures (b.1), (b.2), (b.3), (b.4) and (b.5) and for 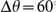 are figures (c.1), (c.2), (c.3), (c.4) and (c.5). When 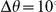, the filament is observed to anchor to the obstacle. Initially it remains roughly straight as shown in (a.1), but with time, it exhibits bending in the transmural direction, without twisting or breaking up. When 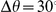, the filament anchors to the obstacle; eventually it bends murally as well as transmurally, twisting around the obstacle but without breaking up. When 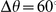, filament breakage occurs in the transmural direction. The broken fragments of the filament remain pinned to the obstacle, but twist around it over and over again. Scroll-wave break-up does not occur away from the obstacle, which explains why our time-series data do not show chaotic signatures.

**Figure 12:**
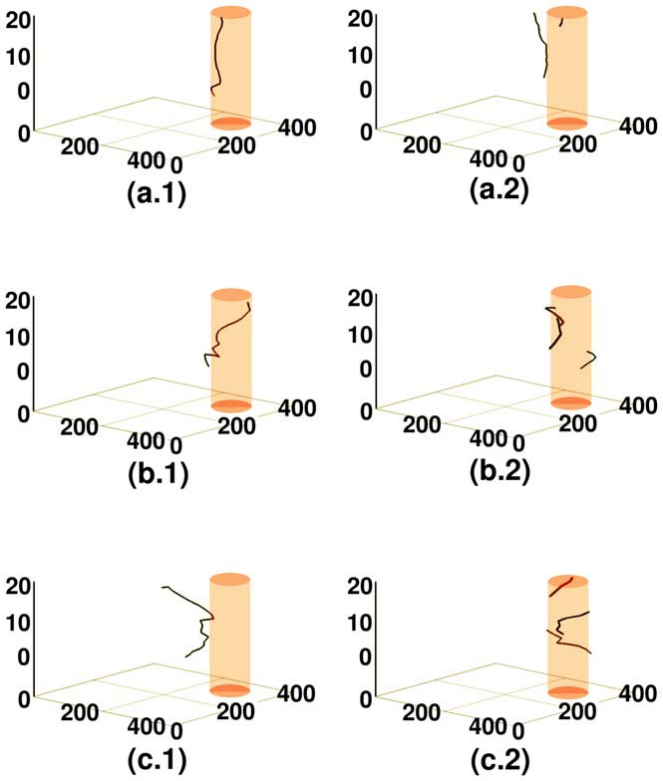
Spacetime evolution of the filament of the scroll wave in the presence of a cylindrical, conduction-type inhomogeneity located at a corner of the simulation domain that was initially close to the core of the scroll wave. These simulations use 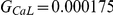 nS/pF. For 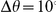, figures (a.1), and (a.2) show, respectively, the scroll- wave-filament at 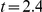
s, and 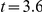
s; the analogs of these figures for 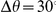 are figures (b.1) and (b.2), and for 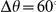 are figures (c.1) and (c.2). When 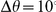, the filament is anchored to the obstacle initially; as time passes it detaches from the obstacle over a considerable fraction of its length. A very small fragment is seen to break off from an end of the filament. When 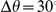, the filament is anchored to the obstacle in some layers but not in others. With time, it breaks up transmurally into smaller filaments, which in turn remain attached to the obstacle. When 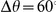, the filament remains detached from the obstacle over a considerable fraction of its length. With the passage of time, breakage occurs in the transmural direction; the broken fragments of the filament get pinned to the obstacle. Scroll-wave break-up does not occur away from the obstacle, which explains why our time-series data do not show chaotic signatures.

Spatiotemporal evolution of the filament of the scroll wave in the presence of a cylindrical, conduction-type inhomogeneity located at a corner of the simulation domain, which is initially far from the core of the scroll wave, is illustrated in figure 10. For 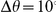, figures 10(a.1), (a.2), (a.3), (a.4) and (a.5) show, respectively, the filament of the scroll wave at 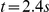, 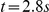, 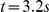, 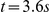, and 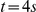; the analogs of these figures for 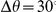 are figures 10 (b.1), (b.2), (b.3), (b.4), and (b.5), and for 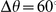 are figures 10 (c.1), (c.2), (c.3), (c.4), and (c.5). In all of the above cases, the presence of the obstacle does not affect the dynamics of the scroll wave. The filament, that is located away from the obstacle in each case, displays bending and twisting, but no breakage. Figure 11 illustrates the spatiotemporal evolution of the filament of the scroll wave in the presence of a cylindrical, conduction-type obstacle, located at the center of the simulation domain; for 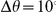, figures 11 (a.1), (a.2), (a.3), (a.4) and (a.5) show, respectively, the filament of the scroll wave at 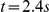, 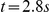, 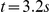, 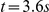 and 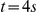. The analogs of these figures for 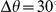 are figures 11 (b.1), (b.2), (b.3), (b.4) and (b.5) and for 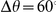 are figures 11(c.1), (c.2), (c.3), (c.4) and (c.5). When 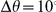, the filament is observed to anchor to the obstacle. Initially it remains roughly straight as shown in 11(a.1), but with time, it exhibits bending in the transmural direction, without twisting or breaking up. When 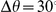, the filament anchors to the obstacle. With time, it bends murally as well as transmurally, twisting around the obstacle but without breaking up. When 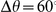, filament breakage occurs in the transmural direction. The broken fragments of the filament remain pinned to the obstacle, but twist around it over and over again. Scroll-wave break-up does not occur away from the obstacle, which explains why our time series data do not show chaotic signatures. Figure 12 illustrates the dynamics of the filament of the scroll wave when the cylindrical, conduction-type obstacle is located at a corner of the simulation domain that was initially close to its core. For 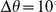, figures 12(a.1), and (a.2) show, respectively, the scroll-wave-filament at 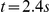, and 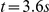; the analogs of these figures for 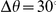 are figures 12 (b.1) and (b.2), and for 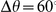 are figures 12(c.1) and (c.2). When 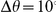, the filament is observed to anchor to the obstacle initially; with time it detaches from the obstacle over a major part of its length. A very small fragment is seen to break off from an end of the filament. When 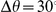, the filament is anchored to the obstacle in some layers but not in others. With time, it breaks up transmurally into smaller filaments, which in turn remain attached to the obstacle. When 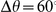, the filament remains detached from the obstacle over a major portion of its length. Gradually with time, breakage occurs in the transmural direction; the broken fragments of the filament get pinned to the obstacle. Scroll-wave break-up does not occur away from the obstacle, which explains why our time-series data do not show chaotic signatures.

**Figure 13:**
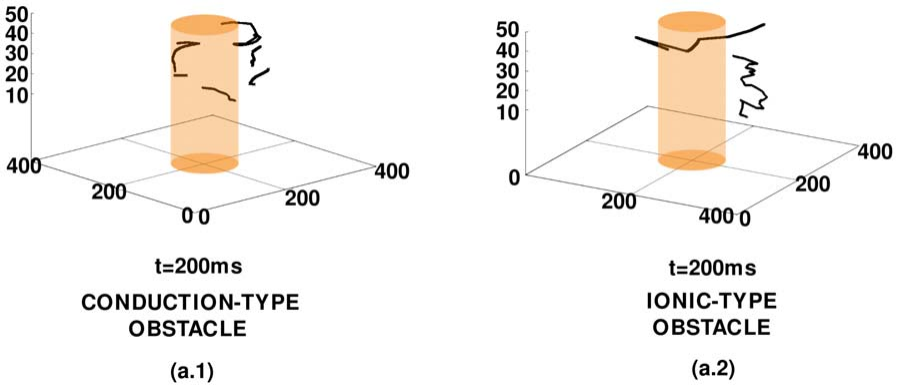
Scroll-wave filament in the 3D TNNP model with transmural heterogeneity and fiber rotation and cylindrical inhomogeneities. Representative plots, at time 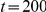
ms of the filament of a scroll wave in the 3D TNNP with transmural heterogeneity and fiber rotation; at 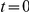 we start with a scroll wave whose filament is straight; the widths of the epicardial, mid-myocardial, and endocardial layers are, respectively, 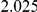 mm, 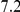 mm, and 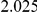 mm; the cylindrical inhomogeneities have radii and heights of 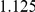 cm and are located at the center of the simulation domain; left and right panels show conduction and ionic inhomogeneities, respectively.

If we now include transmural heterogeneity, as described above, and also place a cylindrical conduction inhomogeneity at the center of the simulation domain, a scroll wave, with a filament that is straight initially, breaks up completely in the transmural direction. The wave still remains anchored to the obstacle along its length at various points; but the differences in wave-propagation speeds in the epicardial, mid-myocardial, and endocardial layers lead to distortion and transmural-breakage of the scroll-wave filament. A representative filament plot for this case is shown in figure 13(a.1).

### Strong-meander régime

We now turn to studies of scroll-wave dynamics in the strong-meander régime of the 3D TNNP model with conduction inhomogeneities. The value of 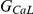 is reduced to one-fourth of its maximal value; and the cylindrical conduction-type inhomogeneity has a radius 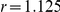 cm.

**Figure 14:**
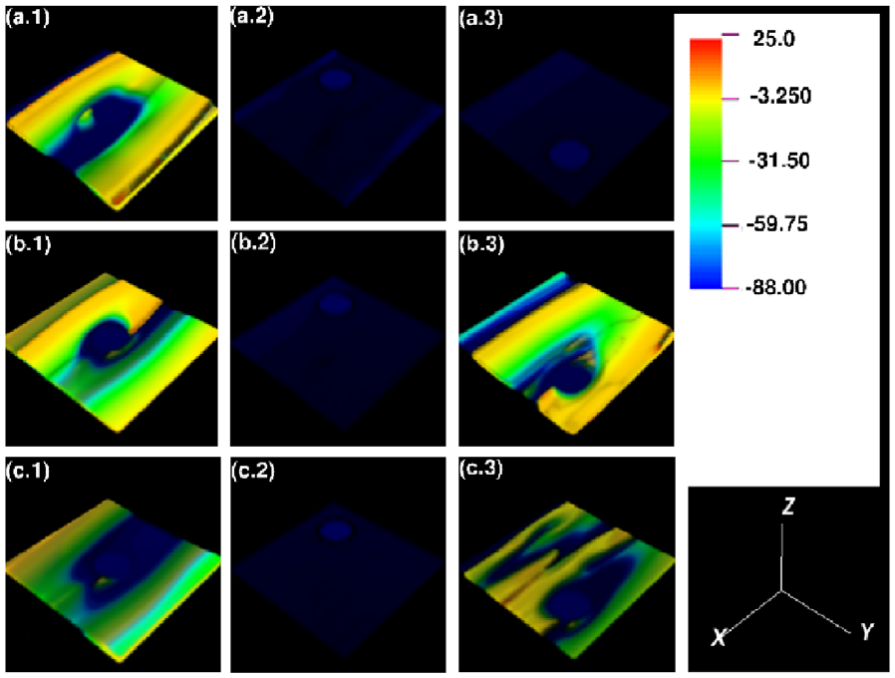
Scroll-wave dynamics in the 3D TNNP model with fiber rotation and a cylindrical conduction inhomogeneity with radius 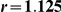 cm and the *L-type* 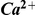 conductance 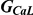 reduced to 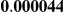 nS/pF from its normal value 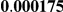 nS/pF. Obstacle at the center of the domain: (a.1) A representative volume rendering of 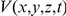, with 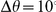, showing broken elements of the scroll wave pinned to the obstacle and the formation of a *seed wave*. (b.1) A representative volume rendering of 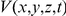, with 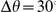, showing some sections of the scroll wave anchoring to the obstacle in some layers but leaving the domain in other layers and a seed wave (the spatiotemporal evolution of 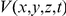 for this case is shown in Video S9 of the supplementary material, which is also available at http://www.physics.iisc.ernet.in/~rahul/new_movies.html). (c.1) A representative volume rendering of 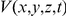, with 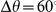, showing wave-pinning and eventual wave-elimination from most of the layers and a seed wave regenerating a single rotating scroll wave. Obstacle at a corner, far from the core of the scroll: the scroll wave impinges on the inhomogeneity, swirls around it, and is then absorbed by the boundaries of the simulation domain as shown in (a.2), (b.2), and (c.2) for 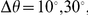 and 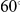, respectively (the spatiotemporal evolution of 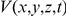 for the representative case of 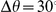 is shown in Video S10 in the supplementary material). Obstacle at a corner that is close to the core of the scroll wave: the wave leaves the simulation domain completely when 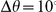 as shown in (a.3); if 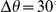, the final state consists of a single rotating scroll wave in a fraction of the medium and the rest of the medium remains free of scroll-wave activity as illustrated in (b.3); if 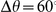, the final state comprises a single rotating scroll wave that wanes periodically and is then regenerated by a seed wave as shown in (c.3) (the spatiotemporal evolution of 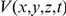 for the representative case of 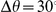 is shown in Video S11 in the supplementary material; the videos for each of the cases illustrated in this figure, are available at http://www.physics.iisc.ernet.in/~rahul/new_movies.html).

We begin with the obstacle at the center of the domain. If 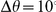, scroll-wave break up occurs close to the obstacle, broken elements of the scroll wave get pinned to the obstacle, whereas parts of the scroll wave that lie away from the obstacle get annihilated by colliding with each other or they move out of the simulation domain. A small fragment of the scroll wave, which we refer to as a *seed wave*, breaks off from a region near the core and eventually regenerates a scroll wave; this cycle of the near elimination and subsequent regeneration of the scroll wave is then repeated over and over again. This is illustrated in the representative volume rendering of 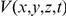 in figure 14(a.1) at the instant of time at which the seed wave forms. Figure 14(b.1) displays a volume-rendering plot of the simulation domain (for 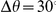), at the instant of seed-wave formation during the spatiotemporal evolution of the scroll wave in the presence of a cylindrical, conduction-type heterogeneity at the center of the domain. We find that, if 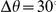, some layers of the domain show sections of the scroll wave anchoring to the obstacle; in other layers the section of the scroll wave is eliminated. Thus, filament- breakage occurs prominently in the transmural direction. The spatiotemporal evolution of 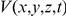 for this case is shown in Video S9 of the supplementary material and also at http://www.physics.iisc.ernet.in/~rahul/new_movies.html. If we increase 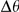 to 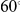, pinning still occurs but for a shorter duration of time than in the cases 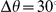 and 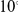. Parts of the scroll wave are eliminated from most of the layers; this leads to the break up of the scroll-wave filament along the transmural direction, in addition to the break up in the plane; in a few layers a seed wave regenerates a single rotating scroll wave and this process is repeated again and again. We illustrate this in the representative volume rendering of 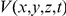 in figure 14(c.1) at the time instant at which the seed wave forms; the spatiotemporal evolution of 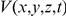 for the representative cases with 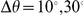 and 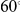 are available at http://www.physics.iisc.ernet.in/~rahul/new_movies.html.

We now place the inhomogeneity near a corner, but far away from the core of the scroll wave. The scroll wave does not get anchored to the inhomogeneity; instead, it impinges on the obstacle, swirls around it, and then is absorbed by the boundaries of the simulation domain. We show this in the representative volume renderings of 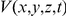 in figures 14(a.2), (b.2), and (c.2) for 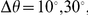 and 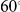, respectively; the spatiotemporal evolution of 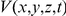 for the representative case of 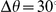 is shown in Video S10 in the supplementary material; the videos for the cases with 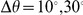 and 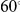 are available at http://www.physics.iisc.ernet.in/~rahul/new_movies.html.

If we locate the obstacle at a corner that is close to the core of the scroll wave, then we find that for 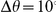 the wave leaves the simulation domain completely. If 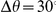, initially the scroll-wave activity is turbulent but the final state consists of a single rotating scroll wave in a fraction of the medium; the rest of the medium remains free of scroll-wave activity (figure 14b.3)). If 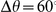, the initial state is a turbulent one with broken-scroll-wave elements; the final state comprises a single rotating scroll wave that wanes periodically and is then regenerated by a seed wave. We show these dynamical behaviors in the representative volume renderings of 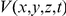 in figures 14(a.3), (b.3), and (c.3) for 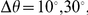 and 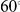, respectively; the spatiotemporal evolution of 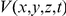 for the representative case of 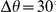 is shown in Video S11 in the supplementary material; the videos for the cases with 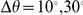 and 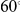 are available at http://www.physics.iisc.ernet.in/~rahul/new_movies.html.

**Figure 15:**
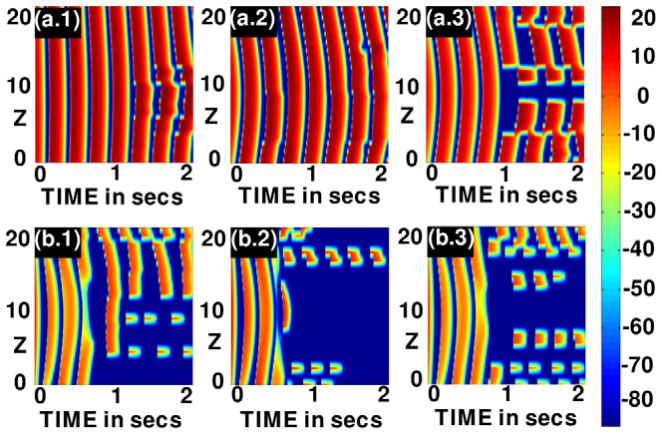
Scroll-wave dynamics in the 3D TNNP model with fiber rotation and a cylindrical conduction inhomogeneity with radius 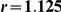 cm. (a.1)-(a.3) illustrate space-time 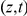 pseudocolor plots of the transmembrane potential 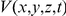 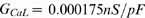
when 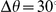 for the original value of 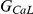 (weak-meander régime) and (b.1)-(b.3) illustrate the same for 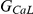 equal to one-fourth of its maximal value (strong-meander régime). In (a.1) and (b.1) the obstacle is at the center of the domain, in (a.2) and (b.2) it is at a corner away from the core of the scroll wave, and in (a.3) and (b.3) the obstacle is at a corner that is close to the scroll-wave core; data for these plots are obtained from the representative point 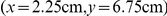.

Space-time 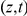 pseudocolor plots of 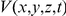 are shown in figures 15(a.1)-(a.3) for the original value of 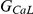 (weak-meander régime) and in figures 15(b.1)-(b.3) for 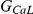 equal to one-fourth of its maximal value (strong-meander régime) and 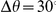; in figures 15(a.1) and 15(b.1) the obstacle is at the center of the domain, in figures 15(a.2) and 15(b.2) it is near a corner but away from the core of the scroll wave, and in figures 15(a.3) and 15(b.3) the obstacle is near the scroll-wave core; data for these plots are obtained the representative point 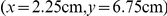.

### Ionic inhomogeneities

We model cylindrical ionic-type inhomogeneities by setting 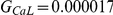 in a cylindrical region of radius 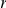; the axis of this cylinder is parallel to the 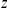 direction; and it spans the thickness of our simulation domain. We present results for 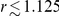 cm.

**Figure 16:**
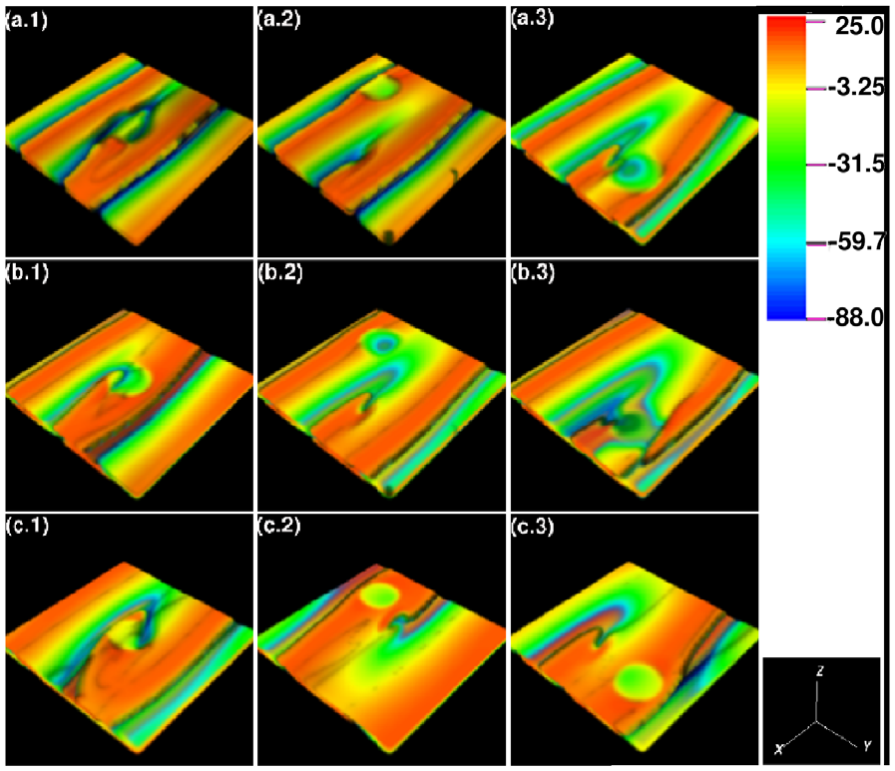
Scroll-wave dynamics in the 3D TNNP model with fiber rotation and a cylindrical ionic inhomogeneity with radius 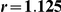 cm. Obstacle at the center of the domain: the scroll wave anchors to the obstacle for 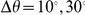, and 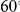 as shown in the representative volume renderings of 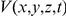 in (a.1), (b.1), and (c.1), respectively (the spatiotemporal evolution of 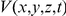 for the representative case of 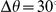 is shown in Video S12 in the supplementary material). Obstacle at a corner far from the core of the scroll: For low 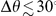, the scroll wave does not get anchored to the inhomogeneity; instead, it impinges on the obstacle, goes around it, reassembles as a plane wave beyond it, and, far from the inhomogeneity, it evolves as it did in the absence of the inhomogeneity. However, if 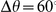, different layers of the simulation domain support sections of the scroll wave that rotate quasi-periodically with different conduction velocities. This is illustrated in (a.2), (b.2), and (c.2) for 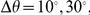 and 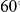, respectively (the spatiotemporal evolution of 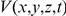 for the representative case of 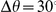 is shown in Video S13 in the supplementary material). Obstacle at a corner that is close to the core of the scroll wave: the wave gets pinned to the inhomogeneity for all the values of 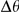 we consider, namely, 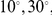 and 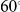 as illustrated in (a.3), (b.3), and (c.3) for 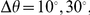 and 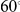, respectively (the spatiotemporal evolution of 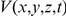 for the representative case of 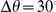 is shown in Video S14 in the supplementary material); the videos for each of the cases illustrated in this figure are available at http://www.physics.iisc.ernet.in/~rahul/new_movies.html).

**Figure 17:**
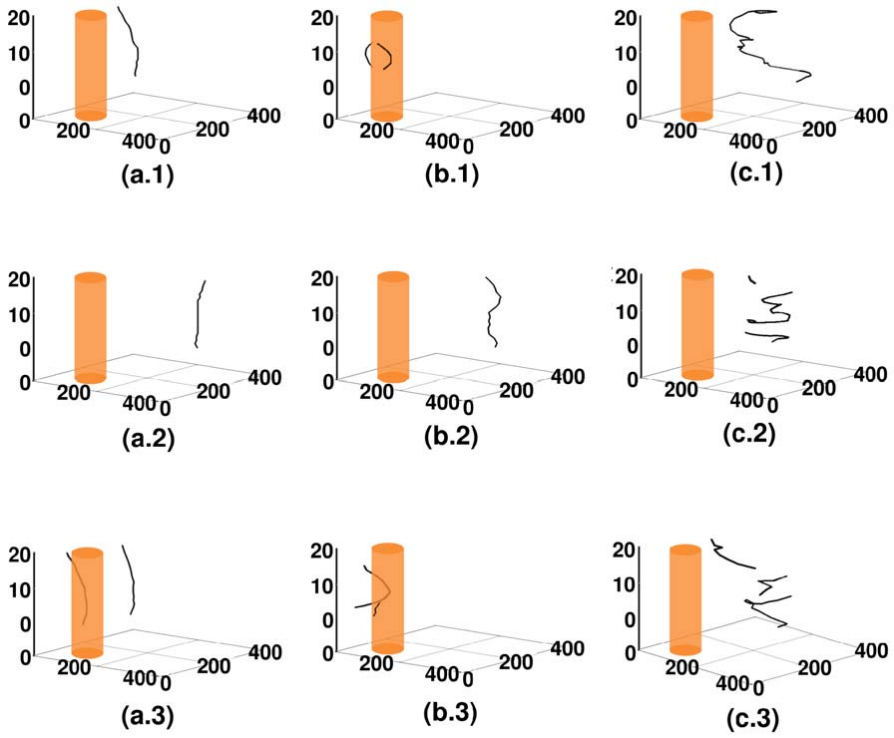
Spacetime evolution of the filament of the scroll wave in the presence of a cylindrical, ionic-type inhomogeneity. Spacetime evolution of the filament of the scroll wave is shown in the presence of a cylindrical, ionic-type obstacle, located at a corner of the simulation domain that was initially far from the core of the scroll wave. For 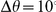, figures (a.1), (a.2), and (a.3) show, respectively, the filament of the scroll wave at 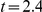
s, 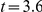
s, and 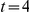
s. The analogs of these figures for 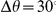 are figures (b.1), (b.2), and (b.3), whereas, for 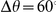, they are figures (c.1), (c.2), and (c.3). When 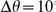, the filament is free from the obstacle initially; it remains unaffected by the presence of the obstacle throughout the duration of the simulation. However, as shown in figure (a.3), at a later time, a second filament is formed within the obstacle; the medium then supports more than one scroll wave. When 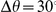, scroll-wave activity is initially supported over a finite region of the domain. Within the region that supports scroll-wave activity, the filament anchors to the obstacle in some layers but not in others. With the passage of time, this filament grows in length, detaches from the obstacle and drifts away from it. At an even later time, the filament breaks up transmurally, enters the obstacle, and remains pinned to it. When 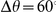, the filament remains detached from the obstacle at all times; initially it is straight; with the passage of time breakage occurs in the transmural direction; the broken fragments of the filament also remain detached from the obstacle.

In the presence of such an ionic-type inhomogeneity we find that the scroll wave anchors to the obstacle for 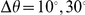, and 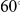, when the obstacle is positioned at the center of the simulation domain. We show these dynamical behaviors in the representative volume renderings of 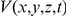 in figures 16 (a.1), (b.1),and (c.1) for 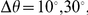 and 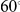, respectively; the spatiotemporal evolution of 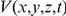 for the representative case of 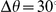 is shown in Video S12 in the supplementary material; the videos for the cases with 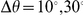 and 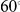 are available at http://www.physics.iisc.ernet.in/~rahul/new_movies.html. These figures and videos show that, in the presence of the ionic inhomogeneity and FR, the dynamical evolution of the scroll wave is such that the system displays spatiotemporal chaos [2], [3] near the location of the inhomogeneity; the rest of the domain supports a scroll wave whose temporal evolution is quasiperiodic. Figure 17 illustrates the spatiotemporal evolution of the filament of the scroll wave, in the presence of a cylindrical, ionic-type obstacle, located at a corner of the simulation domain that was initially far from the core of the scroll wave; for 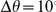, figures 17 (a.1), (a.2), and (a.3) show, respectively, the filament of the scroll wave at 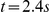, 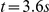, and 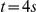. The analogs of these figures for 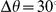 are figures 17 (b.1), (b.2), and (b.3), whereas, for 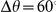 are figures 17(c.1), (c.2), and (c.3). When 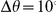, the filament is free from the obstacle initially; it remains unaffected by the presence of the obstacle throughout the duration of the simulation. However, as shown in figure 17(a.3), at a later time, a second filament is formed within the obstacle; the medium then supports more than one scroll wave. When 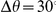, scroll-wave activity is initially supported over a finite region of the domain. The rest of the domain contains plane waves. Within the region that supports scroll-wave activity, the filament is anchored to the obstacle in some layers but not in others. With time, this filament grows in length, detaches from the obstacle and drifts away from it. At an even later time, the filament breaks up transmurally, enters the obstacle and remains pinned to it. When 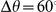, the filament remains detached from the obstacle at all times. Though the filament is initially intact, with time filament breakage occurs in the transmural direction; the broken fragments of the filament also remain detached from the obstacle.

**Figure 18:**
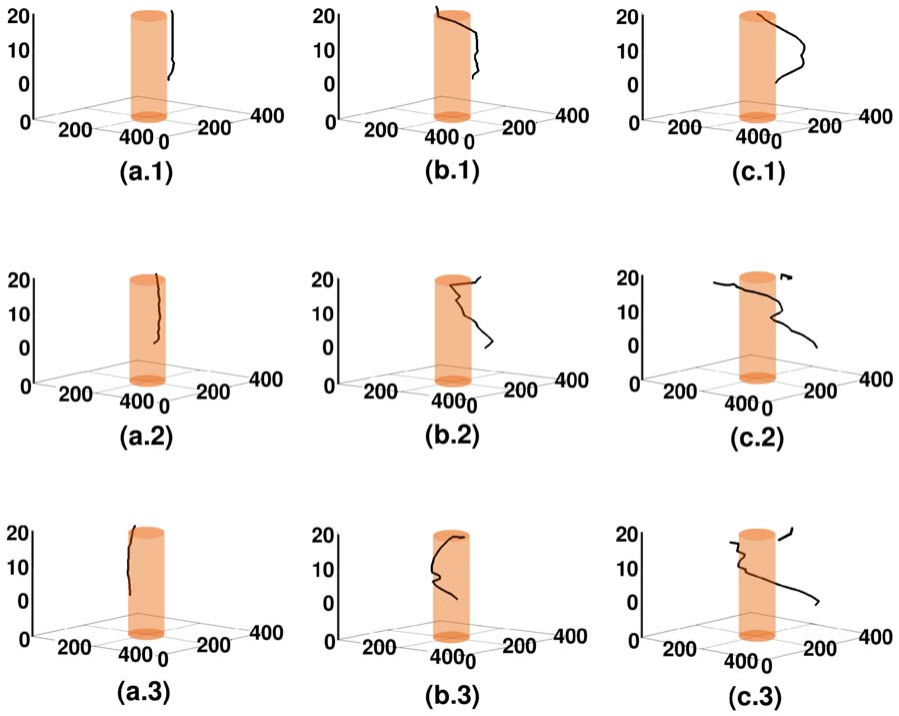
Spacetime evolution of the filament of the scroll wave in the presence of a cylindrical, ionic-type inhomogeneity. Spacetime evolution of the filament of the scroll wave is shown in the presence of a cylindrical, ionic-type obstacle, located at the center of the simulation domain. For 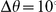, figures (a.1), (a.2), and (a.3) show, respectively, the scroll filament at 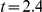
s, 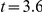
s, and 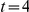
s. The analogs of these figures for 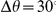 are figures (b.1), (b.2), and (b.3), and for 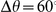, they are figures (c.1), (c.2), and (c.3). When 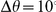, the filament is close to the obstacle initially; with time it attaches to the obstacle and remains pinned to it throughout the duration of the simulation. When 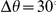, the filament initially twists around the obstacle, without attaching to it. With time, this filament enters the obstacle and anchors to it. When 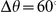, the filament remains twisted around the obstacle at all times, without actually anchoring on to it. Breakage occurs close to the obstacle, in the transmural direction.

**Figure 19:**
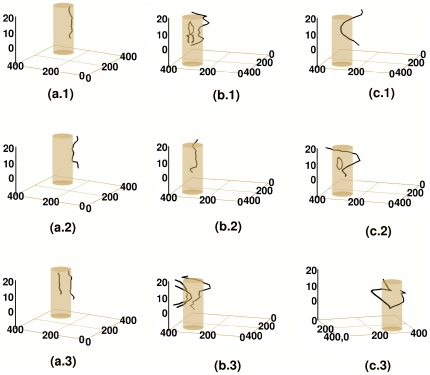
Spacetime evolution of the filament of the scroll wave in the presence of a cylindrical, ionic-type inhomogeneity. Spacetime evolution of the filament of the scroll wave is shown in the presence of a cylindrical, ionic-type obstacle, located at a corner of the simulation domain, that was initially close to the core of the scroll wave; for 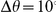, figures (a.1), (a.2), and (a.3) show, respectively, the scroll filament at 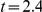
s, 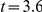
s, and 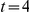
s. The analogs of these figures for 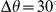, are figures (b.1), (b.2), and (b.3), and for 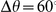 are figures (c.1), (c.2), and (c.3). When 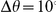, the filament initially anchors to the obstacle, briefly detaches from it, and then anchors again to the obstacle. In the process, a second scroll hook forms within the inhomogeneity, which exists alongside the original filament. When 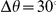, the filament of the scroll wave anchors to the obstacle in some layers but not in others; within the inhomogeneity the filament forms a figure-eight-type structure, which later disappears; the medium now shows more than one scroll-wave filament. When 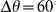, the filament initially bends in the transmural direction, without twisting. Then it enters the obstacle, breaks up, and forms a scroll ring which eventually moves out of the inhomogeneity and merges with the main filament to form a long filament that twists around the obstacle.

The filament of the scroll in the presence of a cylindrical, ionic-type obstacle, located at the center of the simulation domain, is illustrated in figure 18; for 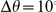, figures 18(a.1), (a.2), and (a.3) show, respectively, the scroll filament at 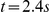, 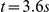, and 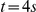. The analogs of these figures for 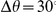 are figures 18(b.1), (b.2), and (b.3), and for 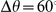 are figures 18(c.1), (c.2), and (c.3). When 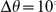, the filament is close to the obstacle initially; with time it attaches to the obstacle and remains pinned to it throughout the duration of the simulation. When 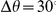, the filament initially twists around the obstacle, without attaching to it. With time, this filament enters the obstacle and successfully anchors to it. When 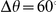, the filament remains twisted around the obstacle at all times, without actually anchoring on to it. Breakage occurs close to the obstacle, in the transmural direction. Spatiotemporal evolution of the filament of the scroll wave in the presence of a cylindrical, ionic-type inhomogeneity, located at a corner of the simulation domain, which was initially close to the core of the scroll wave, is illustrated in figure 19; for 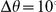, figures 19(a.1), (a.2), and (a.3) show, respectively, the scroll-wave filament at 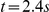, 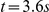, and 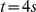. The analogs of these figures for 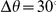, are figures 19(b.1), (b.2), and (b.3), and for 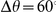 are figures 19(c.1), (c.2), and (c.3). When 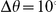, the filament initially anchors to the obstacle, briefly detaches from it and then re-anchors to the obstacle. In the process, a second scroll hook forms within the inhomogeneity, which exists side by side with the original filament. When 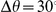, the filament of the scroll wave is anchored to the obstacle in some layers but not in others. The formation of a figure-eight-like structure occurs within the inhomogeneity; this later disappears and the medium acquires the potential to support more than one scroll; hence the existence of three filaments is observed. When 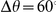, the filament initially bends in the transmural direction, without twisting. Then it enters the obstacle, breaks up and forms a scroll ring which eventually moves out of the inhomogeneity and merges with the main filament to form a long filament that twists angularly around the obstacle.

**Figure 20:**
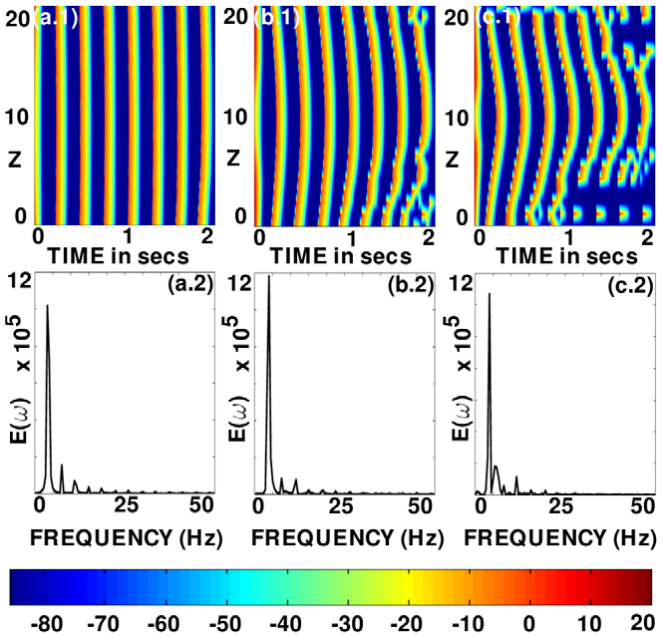
Scroll-wave dynamics in the 3D TNNP model with fiber rotation and a cylindrical ionic inhomogeneity with radius 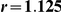 cm. Space-time 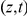 pseudocolor plots of 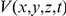 are shown in (a.1), (b.1), and (c.1) at the representative point 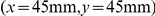 for 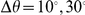, and 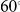, respectively; (a.2), (b.2), and (c.2) show the power spectra 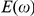 obtained from the time series 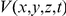 from the representative point 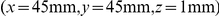, which lies inside the inhomogeneity; the disorder in the space-time plots and the broad-band background in the power spectra, both signatures of spatiotemporal chaos, increase with 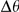.

**Figure 21:**
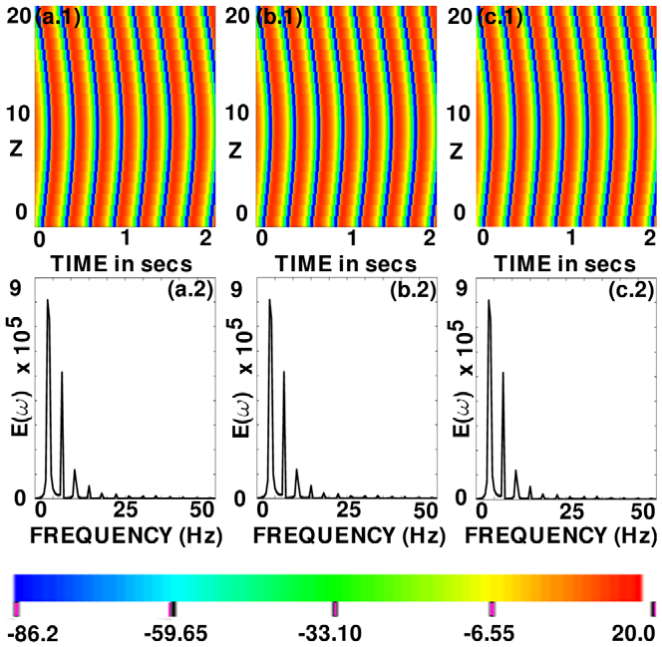
Scroll-wave dynamics in the 3D TNNP model with fiber rotation and a cylindrical ionic inhomogeneity with radius 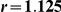 cm. Spacetime 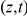 pseudocolor plots of 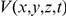 are shown in (a.1), (b.1), and (c.1) at the representative point 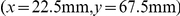, for three positions of the obstacle when 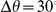. (a.1) shows the 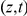 plot for the obstacle at the center of the simulation domain; (b.1) shows the same for the case where the obstacle is at a corner away from the core of the scroll; and (c.1) shows the same for the case where the obstacle is at a corner that is close to the scroll-wave core. There is no indication of spatiotemporal chaos as can be seen from these illustrative space-time plots. Power spectra 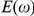 obtained from the time series 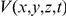 recorded from the representative point 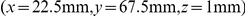 for these three cases are illustrated in (a.2), (b.2), and (c.2); the peaks in these power spectra can be indexed as 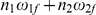, with 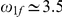 and 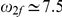 two incommensurate frequencies, so the temporal evolution of the scroll wave, away from the ionic inhomogeneity, is quasi-periodic.

Space-time 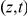 pseudocolor plots of 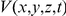 are shown in figures 20 (a.1), (b.1), and (c.1) at the representative point 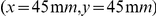 for 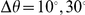, and 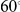, respectively; and figures 20(a.2), (b.2), and (c.2) show the power spectra 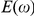 obtained from the time series 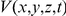 from the representative point 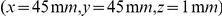, which lies inside the inhomogeneity; the disorder in the space-time plots and the broad-band background in the power spectra, both signatures of spatiotemporal chaos, increase with 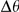. If we record data from a point outside the ionic inhomogeneity then we find no indication of spatiotemporal chaos as can be seen from the illustrative plots, for 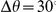, in figures 21(a.1) and 21(a.2); the former is a space-time 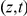 pseudocolor plot of 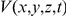 from the representative point 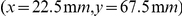 and the latter a power spectrum 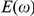 obtained from the time series 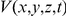 from the representative point 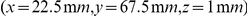; the peaks in this power spectrum can be indexed as 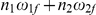, with 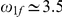 and 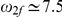 two incommensurate frequencies, so the temporal evolution of the scroll wave, away from the ionic inhomogeneity, is quasi-periodic. This coexistence of spatiotemporal chaos and quasi-periodic behavior in different parts of the simulation domain has been observed earlier in numerical studies of the 2D TNNP model with ionic inhomogeneities [3] but never before in studies of 3D mathematical models for cardiac tissue.

If the ionic inhomogeneity is located near a corner that is away from the core of the scroll wave, for low 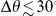, the scroll wave does not get anchored to the inhomogeneity; instead, it impinges on the obstacle, goes around it, reassembles as a plane wave beyond it, and, far from the inhomogeneity, it evolves as it did in the absence of the inhomogeneity. Figure 21(b.1) shows a space-time 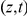 pseudocolor plot of 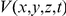 from the representative point 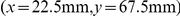 and 21(b.2) is a power spectrum 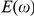 obtained from the time series 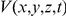 from the representative point 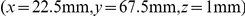; when the ionic inhomogeneity is located near a corner, which is away from the core of the scroll wave, and 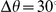. However, if 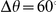, different layers of the simulation domain support sections of the scroll wave that rotate quasi-periodically with different conduction velocities. We show this in the representative volume renderings of 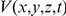 in figures 16(a.2), (b.2), and (c.2) for 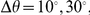 and 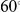, respectively; the spatiotemporal evolution of 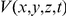 for the representative case of 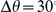 is shown in Video S13 in the supplementary material; the videos for the cases with 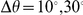 and 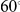 are available at http://www.physics.iisc.ernet.in/~rahul/new_movies.html.

If we place the cylindrical ionic inhomogeneity near a corner of the simulation, which is also close to the core of the scroll wave, we find that the wave gets pinned to the inhomogeneity for all the values of 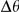 we consider, namely, 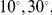 and 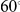; in the case 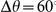 the scroll wave leaves the simulation domain in some layers but continues rotating in other layers. We show this in the representative volume renderings of 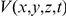 in figures 16(a.3), (b.3), and (c.3) for 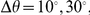 and 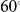, respectively; the spatiotemporal evolution of 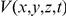 for the representative case of 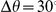 is shown in Video S10 in the supplementary material; the videos for the cases with 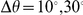 and 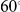 are available at http://www.physics.iisc.ernet.in/~rahul/new_movies.html. Figure 21(c.1) shows the space-time 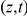 pseudocolor plot of 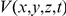 from the representative point 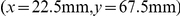 and 21(c.2) shows the power spectrum 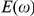 obtained from the time-series 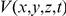 from the representative point 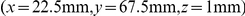; when the ionic inhomogeneity is located near a corner, which is also close to the core of the scroll wave, and 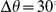.

If we now include transmural heterogeneity, as described above, and also place a cylindrical ionic inhomogeneity at the center of the simulation domain, a scroll wave, with a filament that is straight initially, breaks up transmurally at the boundary between the epicardium and the mid-myocardium. The scroll-wave filament enters the region occupied by the ionic inhomogeneity, leaves it, moves away from it, but always remains close to it. A representative filament plot for this case is shown in figure 19(a.2).

## Discussion

We have presented a systematic study of the combined effects of muscle-fiber rotation and inhomogeneities on scroll-wave dynamics in the TNNP model [44] for cardiac tissue. This model, which has been introduced recently, is based on experimental data obtained from human ventricular cells; it allows for variations of intracellular ion concentrations, contains 12 ionic currents, 12 gating variables, one ion pump, and an ion exchanger; all major ionic currents are included here, e.g., the fast inward 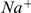 current, the *L-type* 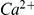 current, the transient-outward potassium current, the slow-potassium-delayed-rectifier current, the rapid-potassium-delayed-rectifier current, and the inward-rectifier 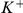 current as described in detail in Ref. [3]. To the best of our knowledge, no other study has attempted to systematize scroll-wave dynamics, in the presence of fiber rotation and inhomogeneities, in such a realistic mathematical model for cardiac tissue. The quantitative results from our study have been described in detail in the previous section. Here we discuss the important qualitative issues that emerge from our study.

We have shown in earlier work [2], [3] that spiral-wave dynamics depends sensitively on the position, shape, and size of inhomogeneities in mathematical models for cardiac tissue; these studies have been restricted to two-dimensional simulation domains, for realistic models like the TNNP model; in three-dimensional simulation domains only illustrative studies have been carried out with inhomogeneities for the simple, two-variable Panfilov model [3], [54]. Our study here extends considerably the results of Refs. [2], [3] by using the three-dimensional TNNP model with fiber rotation. We find, in this case, that, in addition to a sensitive dependence on the positions, shapes, sizes, and types of inhomogeneities, scroll-wave dynamics also depends delicately upon the degree of fiber rotation, which is measured in our calculations by the angle 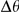.

In one set of studies, we have used parameters, such as the values of certain channel conductances, that are appropriate for the epicardium [44]; whereas, in another set, which contains three representative simulations, we have used parameters that are appropriate for the epicardium, the mid-myocardium as well as the endocardium [44].

In the first set of studies, we have used a slab thickness of 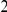 mm because the approximate width of the epicardium in the left ventricle of a human heart is 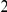 mm (see, e.g., [51]). We have then carried out a study that has never been attempted before, namely, we have elucidated the *combined* effects of fiber rotation and inhomogeneities on scroll-wave dynamics in such tissue. Studies such as those of Refs. [20], [32], [36] have concentrated on the effects of fiber rotation on scroll-wave dynamics *but without inhomogeneities*; our earlier work has elucidated the effects of different types of inhomogeneities –conduction and ionic – on spiral- and scroll-wave dynamics in mathematical models of cardiac tissue *but without fiber rotation*. To the best of our knowledge, the study we present here is the first one to investigate the *combined* effects of fiber rotation and inhomogeneities on scroll-wave dynamics in the detailed ionic TNNP model (principally with epicardial parameters). In the second set of studies, we have included transmural heterogeneity. Hence we have used a simulation domain of total thickness 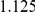 cm.

We have checked in earlier studies (see Ref. [3]) that most qualitative aspects of spiral-wave dynamics in the presence of a conduction inhomogeneity do not depend sensitively on whether we use Neumann boundary conditions on the inhomogeneity or whether we merely set 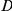 equal to a very small number inside this inhomogeneity; this method of reducing 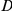 has also been used in Ref. [52]. In this study we are not investigating in detail the interaction of the tip of the spiral wave with the inhomogeneity and the removal of the spiral from such an inhomogeneity by pacing; this might well require a smooth gradient in 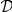 as suggested in Refs. [50], [53].

We find that scroll waves do not anchor easily to cylindrical conduction inhomogeneities with small radii; however, the tendency for anchoring increases with the radius. If parameters such as 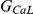 have values that lead to a weak meandering of the scroll wave, then there is a fine balance between anchoring of this wave at the inhomogeneity and a disruption of wave-pinning by fiber rotation; this disruption increases with 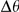. If the parameters are such that the system is in the strong-meander régime, then again the anchoring is suppressed as we increase 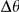; furthermore, the scroll wave can be eliminated from most of the layers only to be regenerated by a seed wave over and over again. Ionic inhomogeneities can also lead to an anchoring of the scroll wave; again fiber rotation works against this anchoring tendency. Scroll waves cannot enter the region inside a conduction inhomogeneity but they can enter the region inside an ionic inhomogeneity; thus, in the latter case, we obtain far richer scroll-wave dynamics than in the former. In particular, we can get a coexistence of spatiotemporal chaos and quasi-periodic behavior in different parts of the simulation domain; this has not been noted before in studies of 3D mathematical models for cardiac tissue. In the simple, conventional picture persistent arrhythmias arise because of the presence of heterogeneities that act as pinning centers; thus, cardiac tissue with a high density of large, in-excitable obstacles is more likely to exhibit VT than tissue that does not. This simple picture may have to be revised in the light of our study.

The formation of a seed wave takes place only when three conditions are simultaneously satisfied: (a) the model parameters are such that the system lies in the strong-meander regime; (b) fiber rotation is present; and (c) the scroll wave interacts with an obstacle whose radius is large enough to anchor it. Given these conditions, if we look near the filament of the scroll wave, we find that the waveback is not smooth: it develops bumps whose 2D analogs have been noted for the LRI model in Refs. [16], [36]. In this 2D study the conduction velocity near the tip varies between fast and slow phases; our 3D study exhibits a similar phenomenon near the scroll-wave filament as it meanders. When it encounters an obstacle, the filament impinges on it and tries to anchor there but the oscillations of the conduction velocity near the core are significantly large; the slow parts of the wave (representing the excitations in the part of the tissue that is relatively refractory) are snapped off the parent wave, while it is attached to the obstacle; this part that has been snapped off is the seed wave.

Major advances in biomedical engineering have found substantial applications in the clinical diagnosis and treatment of cardiac arrhythmias and the electro-physiological study of waves of electrical activation in cardiac tissue and their role in arrhythmias. However, the methods that are used to record such waves in experiments on cardiac tissue are typically constrained to probe only the surface of the tissue. Our *in silico* study of scroll waves in the realistic 3D TNNP model has allowed us to elucidate, by detailed three-dimensional visualization, the effects of inhomogeneities and fiber rotation on the dynamics of these waves. Such visualization is not easy in experiments on three-dimensional cardiac-tissue preparations. Thus, our studies offer insights that complement those derived from experimental measurements [17], [55]–[60] of spiral and scroll waves in cardiac tissue.

The development of a detailed understanding of the influence of fiber rotation and inhomogeneities on scroll-wave activity in cardiac tissue has at least two clinical implications. Such an understanding is important in developing efficient, low-amplitude defibrillation schemes [2], [3], [61], [62]. Furthermore, clinicians should eventually be able to assess the risk of cardiac arrhythmias in different patients from fiber-orientation data, obtained from diffusion-tensor magnetic-resonance imaging (DTMRI).

There are a few limitations of our study: We do not use a bi-domain model, and we do not use an anatomically realistic simulation domain. We conjecture that neither one of these limitations affect the qualitative results of our study; in particular, we expect that, even if we use a bi-domain version of the TNNP model and an anatomically realistic simulation domain, the sensitive dependence of scroll-wave dynamics on inhomogeneities and fiber rotation will persist. Also, recent work [63] has shown that the differences between mono-domain and bi-domain mathematical models for cardiac tissue are not significant in the absence of large electrical stimuli.

Representative *in silico* studies of transmural heterogeneities in 2D, without the types of obstacles we concentrate on and without fiber rotation, can be found in Refs. [41]–[43]. The inclusion of transmural heterogeneity, in three-dimensional simulation domains with fiber rotation, is of importance in developing a detailed understanding of scroll-wave dynamics in ventricular tissue; it has been studied explicitly and systematically for the TNNP model in Ref. [35] but without the types of inhomogeneities we consider here. To perform a detailed, systematic, study of scroll-wave dynamics in the TNNP model, with fiber rotation, transmural heterogeneity and different types of inhomogeneities, such as the ones we have given above, lies beyond the scope of this paper. However, we present three representative results from our simulations on the 3D TNNP model with transmural heterogeneity, fiber rotation, both with and without conduction and ionic inhomogeneities; such studies are computationally expensive.

## Methods

We use the TNNP model [44] of human cardiac tissue for our *in silico* studies. This model is defined by the following reaction-diffusion equation for the transmembrane potential 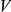: 
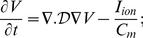
(1)here 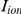 is the total ionic current density, which is expressed as a sum of six major and six minor ionic currents: 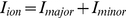
(2)

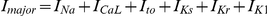


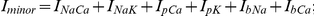
here 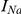 is the fast inward 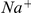 current, 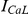 the 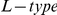 slow-inward 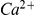 current, 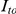 the transient outward current, 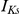 the slow, delayed-rectifier current, 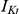 the rapid, delayed-rectifier current, 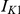 the inward rectifier 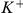 current, 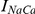 the 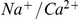 exchanger current, 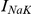 the 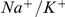 pump current, 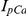 the plateau 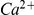 current, 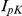 the plateau 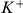 currents, 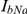 the background 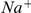 current, and 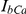 the background 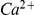 current. Here time 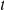 is in milliseconds, voltage 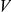 in millivolts, current densities 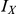 in picoamperes per picofarad (pA/pF), conductances (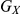) in nanoSiemens per picofarad (nS/pF), and the intracellular and extracellular ionic concentrations (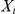, 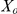) in millimoles per liter (mM/L). When we write 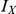, we implicitly mean the total current density per unit capacitance density, because we measure all ionic currents as current density per unit capacitance per unit area as in second-generation models (see, e.g., Refs. [44], [64]–[66]). The capacitance density is measured in units of pF/cm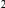, so the unit of 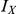, as we have used it, is (pA/cm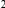) per (pF/cm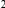), which is nothing but pA/pF. Area and capacitance are related because the specific capacitance of cardiac tissue is of the order of 1
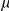
F/cm 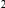. For a detailed list of the parameters of this model and the equations that govern the spatiotemporal behaviors of the transmembrane potential and currents here, we refer the reader to Refs. [3], [44].

In our numerical simulations of the TNNP model we use a three-dimensional domain that is a rectangular slab of dimension 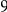 cm 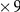 cm 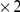 mm. We use a finite-difference method in space with grid spacings 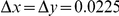 cm and 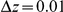 cm and a seven-point stencil for the Laplacian; for time marching we use an Euler method with a time step 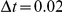
ms. We model cardiac-muscle-fiber orientation as in Refs. [32], [67]: The simulation domain is viewed as a stack of planes with fibers arranged parallel to each other in any given plane. The fibers in each plane are rotated, with respect to those in adjacent planes, by an angle 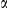; thus, the orientation of the fibers in a plane is specified by an angle 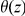 that varies continuously with the transmural coordinate 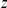. In a normal human heart the average total FR, from the endocardium to the epicardium, is 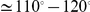. The distribution of this angle over the three-layered heart wall varies across mammalian species and, within a specie, from individual to individual. In our study we consider, in most cases, only a patch of tissue from the epicardium, so we use a smaller value of this average total FR, which we denote by 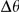. We vary 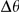 to analyze the effects of FR on the dynamics of scroll waves in the TNNP model for cardiac tissue, both with and without inhomogeneities. We incorporate the effects of FR in this model by using a diffusion tensor 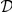 of the form used in Refs. [32], [36]; this has the following components:



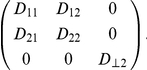



Here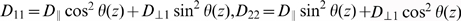 and 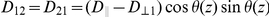. 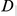 is the diffusivity for propagation parallel to the fiber axis, 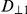 the diffusivity perpendicular to this axis but in the same plane, and 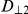 the diffusivity perpendicular to the fiber axis but out of the plane, i.e., in the transmural direction. 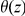, the twist angle along the transmural direction, can expressed as 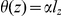, where 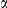 is the rate of fiber rotation and 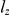 the distance of a given layer from the bottom-most layer of the slab of tissue. We assume that 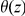 changes continuously in such a way that the total fiber rotation across the tissue is 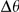. In our studies we use 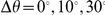 and 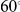. We use 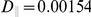 cm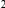/ms and 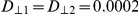 cm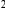/ms.

The initial condition is generated by stacking spiral waves, obtained from 2D simulations on the TNNP model [3], one on top of the other along the 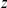 axis, so as to generate a simple scroll wave with a straight filament. The system is then allowed to evolve in time *(i)* in the absence of and *(ii)* in the presence of localized heterogeneities.We then record the spatiotemporal evolution of the transmembrane potential 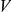.

We consider two types of inhomogeneities: (a) the first is a conduction inhomogeneity; (b) and the second is an ionic inhomogeneity. The former can arise because of an inactive clump of dead cells or scar tissue. Ionic inhomogeneities can arise from functional blockage and the presence of deposits. We model case (a) by introducing a solid, isopotential cylinder of radius 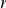 and voltage 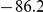 mV at different positions in the simulation domain; the axis of the cylinder is chosen to be along the 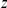 direction; and all elements of the diffusion tensor are assigned very small values (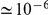 cm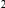 /ms) within the cylinder. The radii of these cylinders are varied. In our simulations we have used 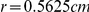 to model small obstacles and 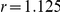 cm to model large obstacles. To model the ionic inhomogeneities in case (b) we modify the value of 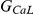 in similar, but not isopotential, cylindrical regions.

We can also include the effects of transmural heterogeneity in the TNNP model. This requires a thicker simulation domain than the one we have used above. The typical thickness of the human ventricular wall is 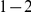 cm and the total fiber rotation 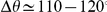. This wall is divided into three layers, namely, the epicardium, the mid-myocardium, and the endocardium. The boundary between the epicardium and the mid-myocardium is sharp, but that between the endocardium and the mid-myocardium is irregular; in our simulations we do not account for such irregularity. We divide our simulation domain into three slabs placed one on top of the other; the thicknesses of the epicardial, mid-myocardial, and endocardial slabs are chosen to be 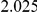 mm, 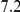 mm, and 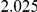 mm, respectively; for each one of these slabs we use parameters, such as the transient outward channel conductance 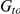, that are appropriate for epicardial, mid-myocardial, and endocardial regions; the values of these parameters are given in Ref. [44]. We have performed three representative simulations with such transmural heterogeneity: (a) in the first we do not have any localized inhomogeneities; (b) in the second we include a cylindrical, conduction-type inhomogeneity; and (c) in the third we introduce a cylindrical, ionic-type inhomogeneity; for cases (b) and (c) the cylindrical inhomogeneities, with radii and heights of 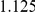 cm, are placed at the center of the simulation domain. Our simulations with transmural heterogeneity show more complex intramural filament shapes and broken filaments than those we observe for scroll-wave filaments in thin epicardial simulation domains. Transmural heterogeneity is perhaps the principal cause for this increase in complexity; but the increase in the thickness of the simulation domain and the degree of fiber rotation are also important. Detailed simulations, which lie beyond the scope of this paper, will have to be designed to see whether the effects of transmural heterogeneity, tissue thickness, and fiber rotation on scroll-wave dynamics can be disentangled from each other.

Furthermore, in order to obtain scroll-wave break up in the TNNP model, with fiber rotation (and/or inhomogeneities), we reduce the Calcium channel conductance, 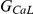 to one-fourth of its original value. This, in effect, blocks 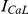. Several experimental and computational studies have shown that altering the value of 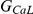 can induce transitions from weak to strong meandering of the tip of a spiral wave as, e.g., in the studies of Refs. [16], [36] for the LRI model. A transition from a single rotating spiral to spiral-wave break up can be obtained by altering other types of ionic conductances also. For example, in our previous studies [3] of the two-dimensional TNNP model of ventricular tissue we have shown that increasing the plateau Calcium conductance 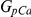 or decreasing the plateau Potassium conductance 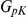 can be responsible for such spiral-wave break-up. Here we change 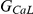 and the total fiber rotation (FR) and study their effects on scroll-wave dynamics in our three-dimensional simulation domain because the Calcium ion channel can be controlled easily by pharmaceutical means as has been done in several studies [68]–[70].

## Supporting Information

Video S1Scroll-wave dynamics in the 3D TNNP model in the absence of fiber rotation and inhomogeneities: An animated volume rendering, illustrating the spatiotemporal evolution of the transmembrane potential 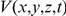, for the same parameter values as in figure 1 (a), with 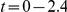
s and 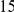 frames per second.(MPEG)

Video S2Scroll-wave dynamics in the 3D TNNP model in the absence of inhomogeneities, but in the presence of fiber rotation with 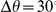: An animated volume rendering, illustrating the spatiotemporal evolution of the transmembrane potential 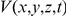, for the same parameter values as in figure 2(c), with 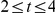
s and 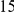 frames per second.(MPEG)

Video S3Scroll-wave dynamics in the 3D TNNP model in the absence of inhomogeneities, but in the presence of fiber rotation with 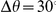: An animated pseudocolor rendering, illustrating the spatiotemporal evolution of the transmembrane iso-potential 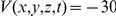 mV, for the same parameter values as in figure 2 (c), with 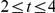
s and 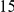 frames per second.(MPEG)

Video S4Scroll-wave dynamics in the 3D TNNP model in the absence of inhomogeneities, but in the presence of fiber rotation with 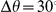 and the 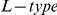 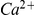 conductance 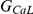 reduced to 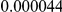 nS/pF from its normal value 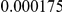 nS/pF: An animated pseudocolor rendering, illustrating the spatiotemporal evolution of the transmembrane potential 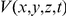, for the same parameter values as in figures 3 (b.1)-(b.4), with 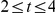
s and 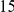 frames per second.(MPEG)

Video S5Scroll-wave dynamics in the 3D TNNP model in the absence of inhomogeneities, but in the presence of fiber rotation with 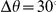 and the 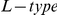 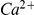 conductance 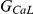 reduced to 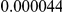 nS/pF from its normal value 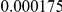 nS/pF: An animated pseudocolor rendering of the 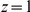 mm layer of the simulation domain, illustrating the spatiotemporal evolution of the transmembrane potential 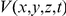 for the same parameter values as in figures 3 (b.1)-(b.4), with 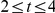
s and 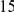 frames per second.(MPEG)

Video S6Scroll-wave dynamics in the 3D TNNP model in the presence of fiber rotation 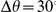 and a cylindrical conduction inhomogeneity of radius 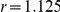 cm, located at the center of the simulation domain: An animated volume rendering, illustrating the spatiotemporal evolution of the transmembrane potential 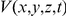, for the same parameter values as in figure 5 (b.1), with 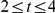
s and 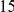 frames per second.(MPEG)

Video S7Scroll-wave dynamics in the 3D TNNP model in the presence of fiber rotation with 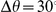 and a cylindrical conduction inhomogeneity of radius 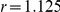 cm, located at a corner of the simulation domain, far from the core of the scroll: An animated volume rendering, illustrating the spatiotemporal evolution of the transmembrane potential 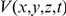, for the same parameter values as in figure 5 (b.2), with 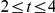
s and 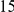 frames per second.(MPEG)

Video S8Scroll-wave dynamics in the 3D TNNP model in the presence of fiber rotation with 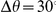 and a cylindrical conduction inhomogeneity of radius 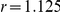 cm located at a corner of the simulation domain that is close to the scroll-wave core: An animated volume rendering, illustrating the spatiotemporal evolution of the transmembrane potential 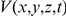, for the same parameter values as in figure 5 (b.3), with 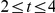
s and 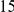 frames per second.(MPEG)

Video S9Scroll-wave dynamics in the 3D TNNP model in the presence of fiber rotation with 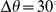 and a cylindrical conduction inhomogeneity of radius 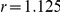 cm located at the center of the simulation domain, and with the 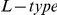 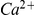 conductance 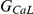, reduced to 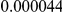 nS/pF from its normal value 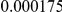 nS/pF: An animated volume rendering, illustrating the spatiotemporal evolution of the transmembrane potential 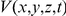, for the same parameter values as in figure 6 (b.1), with 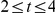
s and 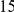 frames per second.(MPEG)

Video S10Scroll-wave dynamics in the 3D TNNP model in the presence of fiber rotation with 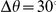 and a cylindrical conduction inhomogeneity of radius 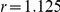 cm located at the corner of the simulation domain, far from the core of the scroll, and with the 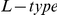 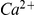 conductance 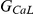, reduced to 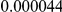 nS/pF from its normal value of 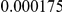 nS/pF: An animated volume rendering, illustrating the spatiotemporal evolution of the transmembrane potential 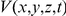 for the same parameter values as in figure 6 (b.2), from 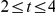
s and with 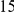 frames per second.(MPEG)

Video S11Scroll-wave dynamics in the 3D TNNP model in the presence of fiber rotation with 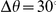, a cylindrical conduction inhomogeneity of radius 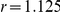 cm located at the corner of the simulation domain that is close to the scroll-wave core, and with the 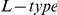 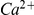 conductance 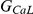, reduced to 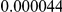 nS/pF from its normal value 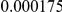 nS/pF: An animated volume rendering, illustrating the spatiotemporal evolution of the transmembrane potential 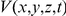, for the same parameter values as in figure 6 (b.3), with 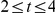
s and 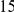 frames per second.(MPEG)

Video S12Scroll-wave dynamics in the 3D TNNP model in the presence of fiber rotation with 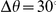 and a cylindrical ionic inhomogeneity of radius 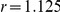 cm located at the center of the simulation domain: An animated volume rendering, illustrating the spatiotemporal evolution of the transmembrane potential 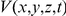, for the same parameter values as in figure 8 (b.1), with 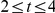
s and 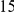 frames per second.(MPEG)

Video S13Scroll-wave dynamics in the 3D TNNP model in the presence of fiber rotation with 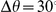 and a cylindrical ionic inhomogeneity of radius 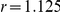 cm located at a corner of the simulation domain far from the core of the scroll: An animated volume rendering, illustrating the spatiotemporal evolution of the transmembrane potential 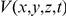, for the same parameter values as in figure 8 (b.2), with 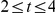
s and 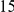 frames per second.(MPEG)

Video S14Scroll-wave dynamics in the 3D TNNP model in the presence of fiber rotation with 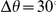 and a cylindrical ionic inhomogeneity of radius 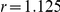 cm located at a corner of the simulation domain that is close to the scroll-wave core: An animated volume rendering, illustrating the spatiotemporal evolution of the transmembrane potential 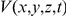, for the same parameter values as in figure 8 (b.3), with 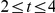
s and 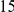 frames per second.(MPEG)
